# Lifestyle patterns influence the composition of the gut microbiome in a healthy Chinese population

**DOI:** 10.1038/s41598-023-41532-4

**Published:** 2023-09-02

**Authors:** Yi Ren, Jiawei Wu, Yilin Wang, Lanying Zhang, Jing Ren, Zhiming Zhang, Binghan Chen, Kejian Zhang, Baoli Zhu, Wei Liu, Sabrina Li, Xu Li

**Affiliations:** 1 Coyote Bioscience (Beijing) Co., Ltd., Beijing, China; 2 Coyote Diagnostics Lab (Beijing) Co., Ltd., Beijing, China; 3 State Key Laboratory of Pathogen and Biosecurity, Beijing Institute of Microbiology and Epidemiology, Beijing, China

**Keywords:** Computational biology and bioinformatics, Microbiology, Health care

## Abstract

High-throughput sequencing allows for the comprehensive analysis of the human intestinal microbiota. However, extensive association analyses between the microbiome and lifestyle differences in the Chinese population are limited. Here, we carried out an independent cohort study—the Chinese Healthy Gut Project (*n* = 483)—where correlations between the gut microbiota and dietary and lifestyle variables in a healthy Chinese population are defined. We collected both questionnaire data, including basic information and lifestyle and dietary variables, and fecal stools from the enrolled volunteers. We then performed 16S rRNA sequencing on the microbial DNA isolated from the stools to assess the composition of the intestinal microbiota. We found that *Prevotella* and *Bacteroides* were the most abundant genera in the healthy Chinese gut microbiome. Additionally, 9 out of 29 clinical and questionnaire-based phenotype covariates were found to be associated with the variation in the composition of the gut microbiota. Among these lifestyle phenotypes, sleep procrastination, negative mood, and drinking habits had the largest effect size. Additionally, an appreciable effect of urbanization was observed, resulting in decreased intra-individual diversity, increased inter-individual diversity, and an increased abundance of the *Bacteroides* enterotype. The results of this study provide a foundation for assessing the healthy Chinese gut microbiota community structure at baseline in a healthy Chinese population. Furthermore, this study also provides insights into understanding how distinctive living habits influence the relationships between the Chinese gut microbiome and systemic health state.

## Introduction

The human gastrointestinal tract is home to a diverse and abundant microbial community. More than 100 trillion microorganisms have been reported to reside within the human intestine^1^, with more than 2000 different species^2^. This intestinal microbial community contains symbiotic, commensal, and pathogenic microorganisms^3^, and the number of the microorganisms in the human colon can reach 10^12^–10^14^, rendering it one of the most densely populated microbial habitats^4, 5^. The intestinal microbiome encodes more than three million genes and produces thousands of metabolites, and as such, is an important factor regulating human health^6^. Previous studies have highlighted the diverse roles the gut microbiome plays in host health, including digestion, immune homeostasis, colonization resistance against pathogens, and the production of vitamins and short-chain fatty acids. Disruptions in the composition and function of the gut microbiome have a direct impact on human diseases, such as inflammatory bowel disease^7, 8^, type II diabetes^9^, and cardiovascular diseases^10^. Additionally, several studies have reported that restoring the homeostatic balance to the gut flora may prevent specific diseases by changing the composition and structure of the gut microbiome (reviewed in)^6, 11, 12^.

Previous studies have not only shown that thousands of different microbes may collectively comprise the human gut microbiota but also confirm a high degree of variation in the composition of the intestinal flora between individuals^13–15^. Despite this inter-individual variation in microbial taxa, the abundance of microbial genes responsible for basic metabolic and housekeeping functions are fairly similar between individuals^13, 15^. Numerous studies regarding the composition of the intestinal microbiome have demonstrated that several factors, including geographical location, host genetics, diet, and lifestyle, influence differences across individuals in terms of the diversity, structure, and composition of the gut microbiota^16–22^. Taken together, these studies suggest that the human gut microbiome is influenced by individual lifestyle variables, and understanding the relationships between the gut microbiome and various lifestyles and dietary patterns prior to the onset of disease may help guide disease treatment.

The identification of the distribution of bacterial taxa in populations with different genetic backgrounds and lifestyle patterns may be useful in understanding mechanisms linking lifestyle patterns with overall health or the risk of disease^23–25^. Reproducible patterns of microbial variation—bacterial taxa that can be separated into clusters termed “enterotypes”^26^—have been observed in the adult human gut^26–32^. To date, most population-level studies have described differences and associations in the gut microbiome from individuals with specific diseases or healthy control cohorts in distinct geographic locations, such as Asia, Europe, and the United States of America^18, 20, 21, 26, 33–37^. Although a few studies have investigated the gut microbiota characteristics of China, they do not adequately reflect the gut microbiota of China as a whole, as these studies had limited participants, focused on specific regions, or lacked sufficient lifestyle data^38–41^. Thus, large-scale phenotyping studies that integrate gut microbiome profiles with comprehensive lifestyle phenotypes in the Chinese population remain scarce and are of great significance for a detailed understanding of the characteristics of the Chinese gut microbiome under different sub-health states.

The aim of this study was to compare the gut microbiota in healthy (no apparent diseases) Chinese volunteers (*n* = 483) and to correlate differences in the gut microbiota with various lifestyle variables. We performed 16S rRNA sequencing on stools collected from the enrolled volunteers and correlated the results with the demographic, diet, and lifestyle information provided by the volunteers via a questionnaire. The results of this study provide insights into the intricate interplay between dietary and lifestyle variables and the gut microbiota in a healthy Chinese population.

## Results

### Characteristics and distribution of intestinal flora in healthy people

The data presented in this study were collected from 483 healthy Chinese people. All participants completed a questionnaire regarding basic demographic and lifestyle data. The information collected from the questionnaire is described in the Materials and Methods section and is presented in Table 1. Feces were collected from 483 participants, and the gut microbiomes were assessed using 16S rRNA sequencing. In total, 483 sequencing samples were obtained. The participants spanned 11 ethnic groups, had an average age of 36.96 years, an average body mass index (BMI) of 22.36, were 65.42% female, and were from 62 residential areas (Table 2).

**Table 1:** Lifestyle patterns collected from the questionnaires of 483 healthy Chinese people (*n* = 483).

	Characteristics	n (%)
Bowel habits (4)		
Intestinal symptoms	Constipation	58 (12.0)
	Hematochezia	2 (0.4)
	Abdominal pain	1 (0.2)
	Diarrhea	41 (8.5)
	Abdominal distension	13 (2.7)
	Indigestion	12 (2.3)
	Normal	356 (73.7)
Stool texture/shape	Soft blobs with clear-cut edges	16 (3.3)
	Separate hard lumps, like nuts	15 (3.1)
	Sausage-shaped but lumpy/like a sausage but with cracks on the surface	42 (8.7)
	Watery, no solid pieces, all liquid	1 (0.2)
	Sausage-shaped, smooth and soft	265 (54.9)
	Fluffy pieces with ragged edges, a mushy stool	144 (29.8)
Stool color	Black/brown	135 (28.0)
	Yellow	347 (71.8)
	Blood-stained or red	1 (0.2)
Stool smell	Stinks often	48 (9.9)
	Stinks seldom	188 (38.9)
	Normal	247 (51.1)
Allergens and health (4)		
Allergies	No	392 (81.2)
	Yes	91 (18.8)
Take antibiotics	No	443 (91.7)
	Yes	40 (8.3)
Whether to take probiotics	No	370 (76.6)
	Yes	113 (23.4)
Presence of mouth sores or pimples	Seldom	246 (50.9)
	Often	64 (13.3)
	Occasional	173 (35.8)
Eating habits (5)		
Dietary preference	Balance	319 (66.0)
	Meat	107 (22.2)
	Vegetables	57 (11.8)
Drink	White tea	3 (0.6)
	Water	237 (49.1)
	Juice	24 (5.0)
	Dark tea	2 (0.4)
	Black tea	21 (4.3)
	Green tea	97 (20.1)
	Yoghurt	54 (11.2)
	Sodas	34 (7.0)
	Oolong tea	9 (1.9)
	Herb tea	2 (0.4)
Starch intake	Rice	390 (80.7)
	Flour	57 (11.8)
	Cereals	30 (6.2)
	Corn/sweet potato	6 (1.2)
The frequency of snacking	Never	117 (24.2)
	Often	92 (19.0)
	Seldom	274 (56.7)
Protein intake	Eggs	95 (19.7%)
	Beans	47 (9.7)
	Milk	68 (14.1)
	Meat	273 (56.5)
Other living habits (7)		
Exercise frequency	No	233 (48.2)
	Yes	250 (51.8)
	Yes—once or twice a week	157 (32.5)
	Yes—more than three times a week	93 (19.3)
State of fatigue	Normal	128 (26.5)
	Sometimes	283 (58.6)
	Always	72 (14.9)
Alcohol intake	Never	333 (68.9)
	Seldom	127 (26.3)
	Often	23 (4.8)
Smoking	No	387 (80.1)
	Seldom	44 (9.1)
	Often	52 (10.8)
Sleep deprivation	No	145 (30.0)
	Seldom	207 (42.9)
	Often	131 (27.1)
Mysophobia	No	295 (61.1)
	Seldom	188 (38.9)
Negative emotions	No	286 (59.2)
	Yes	197 (40.8)

**Table 2:** The characteristics of the study participants (*n* = 483).

Characteristics	*n* (%)
Gender	
Female	316 (65.4)
Male	167 (34.6)
Age	
< 20	17 (3.5)
20–30	141 (29.2)
30–40	152 (31.5)
40–50	97 (20.1)
50–60	57 (11.8)
60–70	16 (3.3)
> 70	3 (0.6)
Ethnicity	
Bai	28 (5.8)
Bouyei	1 (0.2)
Korean	2 (0.4)
Hani	1 (0.2)
Han	418 (86.5)
Hui	8 (1.7)
Lisu	1 (0.2)
Manchu	5 (1.0)
Miao	4 (0.8)
Naxi	1 (0.2)
Yi	14 (2.9)
Habitation	
North	128 (26.5)
South	355 (73.5)
BMI	
Underweight	38 (7.9)
Normal weight	352 (72.9)
Overweight	93 (19.2)

BMI body mass index

**Figure 1:**
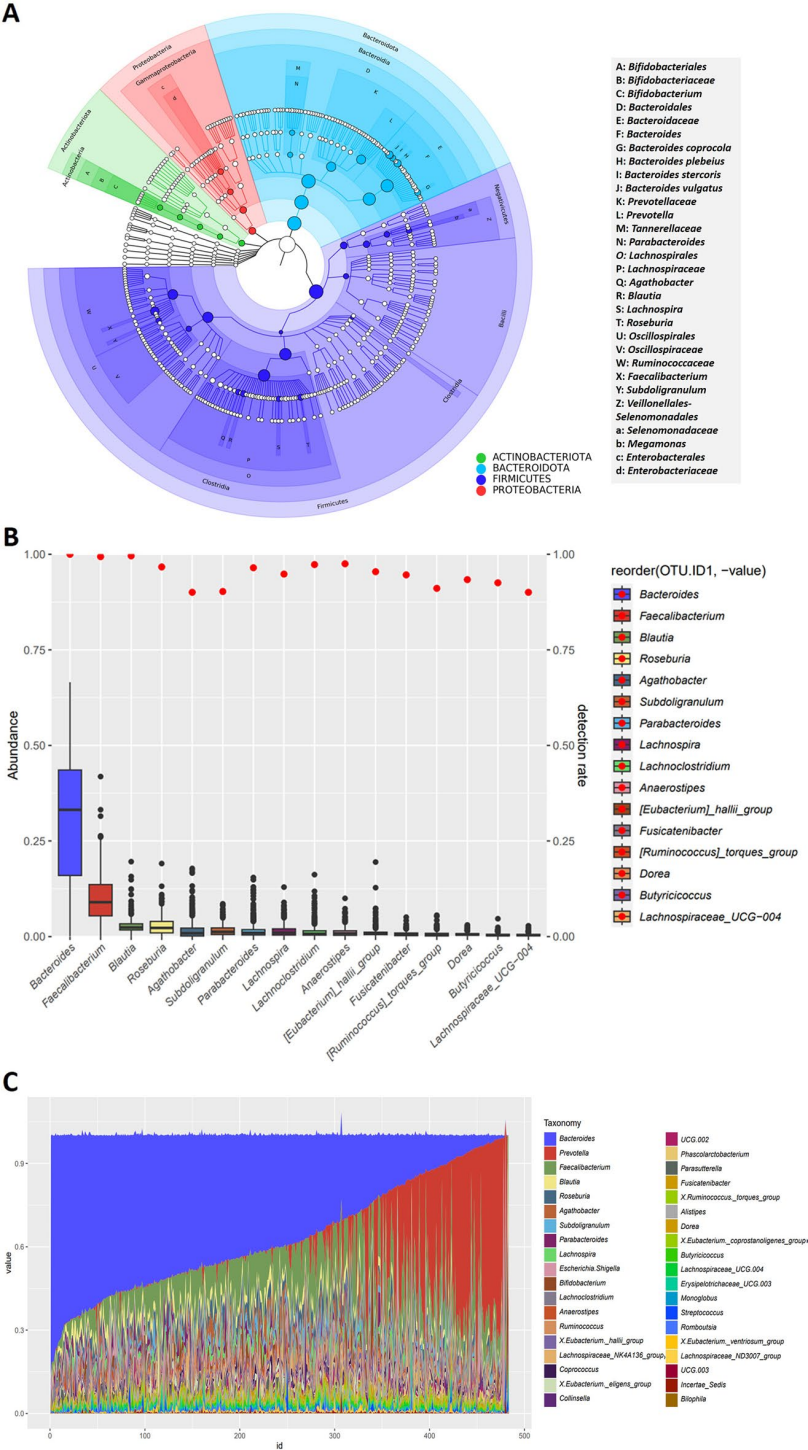
The gut microbiota composition and the high-frequency bacteria of 483 healthy Chinese volunteers. (**A**) GraPhlAn diagram of bacterial genera based on amplicon sequence variants (ASVs annotations). According to these annotations, two phyla, *Bacteriodota* and *Firmicutes*, accounted for the majority of the microbial composition in healthy Chinese volunteers. (**B**) The 16 genera of bacteria were detected in over 90 percent of the samples, with *Bacteriodes* and *Faecalibacterium* exhibiting the highest relative abundance. (**C**) Relative abundances of 38 high-frequency genera in 483 stool samples. Similarly, *Bacteroides* (shown in blue), *Prevotella* (red)*,* and *Faecalibacterium* (green) combined accounted for nearly 80% of the feces of the healthy Chinese people.

A total of 2408 Amplicon Sequencing Variants (ASV) were identified from the 483 samples, i.e., 10 phyla, 15 classes, 34 orders, 61 families, 171 genera. Eighteen genera of bacteria previously reported to be beneficial to humans, including *Bacteroides* (30.38%)^42, 43^, *Prevotella* (11.72%)^44^, and *Faecalibacterium* (9.72%)^45–48^, were identified, while two genera that may exert a negative influence on humans, including *Veillonella* (0.15%)^49^ and *Proteus* (0.002%)^50, 51^, were identified.. When combining the ASV annotation results of all 483 samples, we found that both Proteobacteria and Firmicutes comprised the majority of the microbial composition in healthy Chinese adults (Fig. 1A).

We then grouped the genera based on their detection rate, defined as the sample size of a certain bacterium/total sample size. We divided 171 bacterial genera into groups based on their abundance frequency—low-frequency flora (detection rate < 10%), medium-frequency flora (detection rate 10–70%), and high-frequency flora (detection rate > 70%). After grouping, we identified a total of 38 high-frequency genera, 66 medium-frequency genera, and 67 low-frequency genera. The 16 genera of high-frequency bacteria were detected in over 90% of the samples, indicating their status as fundamental intestinal microorganisms within the Chinese population (Fig. 1B). Notably, eight of these genera were also found in the core gut microbiota of Guangdong province^93^, six were among the top 9 most abundant fecal genera in another Chinese cohort^94^, five overlapped with the top 20 fecal genera discovered in the Human Microbiome Project^95^, and eight were part of the core microbiota in a Chinese cohort comprising 2678 healthy individuals from 28 provinces^96^. And Bacteroides, Blautia and Faecalibacterium were overlapped among our study and the studies above. The 38 high-frequency genera in 483 samples are shown in Fig. 1C.

### Analysis of enterotypes in a healthy Chinese population

**Figure 2:**
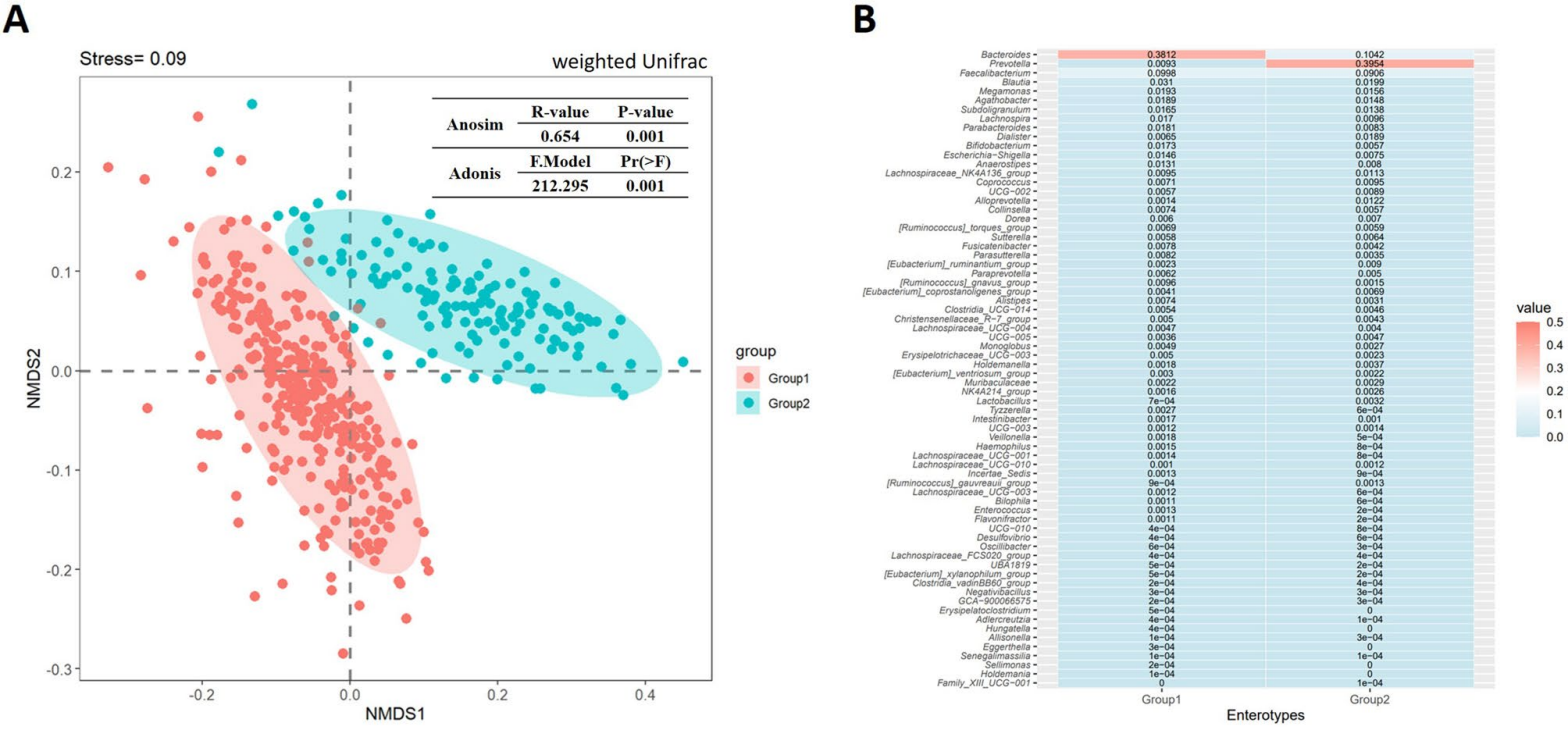
The major enterotypes found in the stool samples from the healthy Chinese population. (**A**) Non-metric multidimensional scaling analysis (NMDS) of the sequencing results from 483 stool samples showed that the intestinal flora in the healthy population of Chinese could be divided into two groups. (**B**) Heatmap of the intestinal flora with significant difference between the two groups in a healthy Chinese population. As shown, *Bacteroides* (38.12%) and *Prevotella* (39.54%) were the driving taxa in Group 1 and Group 2, respectively.

Previous studies have demonstrated that the gut microbiota of various human populations clusters around three primary driver taxa (enterotypes): *Prevotella*, *Bacteroides*, and *Ruminococcus*^26, 27^. In order to analyze the enterotypes of healthy Chinese people, we performed unsupervised clustering on the sequencing results from the 483 stool samples. The clustering results showed that the gut bacteria of healthy Chinese people could be divided into two groups, *Prevotella* (39.54%, n = 135) and *Bacteroides* (38.12%, n = 348) with a significant different gut microbiota structure (weighted unifrac distance, Adonis, Pr(> F) = 0.001; Anosim, *p* = 0.001); a *Ruminococcus* enterotype was not found (Fig. 2A,B, Supplementary Fig. 1, Supplementary Table 1). This is consistent with a previous study, which demonstrated that only *Prevotella* and *Bacteroides* were common enterotypes in Chinese populations^52^. Thus, enterotypes with *Prevotella* and *Bacteroides* as the driving taxa are more common in the Chinese population.

### Demographic factors and Bacteria

The variables of demographic factors including gender, age, BMI, ethnicity and habitation were associated with the composition and structure of the gut microbiota^89^. In this study, we performed differential analysis of the gut microbiota in five demographic factors variables: (1) gender, (2) age, (3) BMI, (4) ethnicity, and (5) habitation.

**Figure 3:**
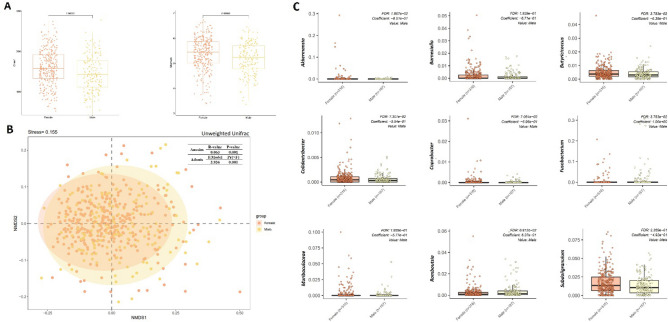
Differences in the microbiota alpha diversity, composition, and bacteria in genus-level between male (n = 167) and female (n = 316). (**A**) Female had higher richness and evenness of gut microiota than male. (**B**) Non-metric multidimensional scaling analysis (NMDS) based on unweighted Unifrac distance matric showed that the two gender groups had separated microbita composition. (**C**) Nine bacteria in genus-level were significantly different between the two gender groups.

Gender was reported as one of the strongest associated factors of gut microbiota structure^89^. In this study, microbiota in female gut (n = 316) showed a distinct pattern in structure and relative abundance in genus-level compared with that in male gut (n = 167). The microbiota richness (Chao 1 index, *p* < 0.001, *q* < 0.001, K–W test) and evenness (Shannon index, *p* < 0.001, *q* < 0.001, K–W test) was significant different between the two gender groups, and female was seemed to have higher richness and evenness of gut microiota. As to beta diversity, clustering by gender was distinguishable on NMDS analysis (weighted unifrac distance, Adonis, Pr (> F) = 0.001; Anosim, *p* = 0.001). 34 bacteria in genus-level showed a significant different between the two gender groups (Fig. 3A,B, Supplementary Table 2). After removing the potential confounding factors, *Akkermansia* (*t*-test, *p* < 0.001, *q* = 0.107; MaAsLin, Coef = -0.851, *p* < 0.001, *q* = 0.019), *Butyricicoccus* (*t*-test, *p* = 0.013, *q* = 0.106; MaAsLin, Coef = -0.638, *p* = 0.004, *q* = 0.038), *Coprobacter* (*t*-test, *p* = 0.027, q = 0.181; MaAsLin, Coef = − 0.596, *p* = 0.012, *q* = 0.071) and *Colidextribacter* (*t*-test, *p* < 0.001, *q* = 0.107; MaAsLin, Coef = − 0.554, *p* = 0.014, *q* = 0.073) were still significant higher in female, while Fusobacterium (*t*-test, *p* = 0.031, *q* = 0.108; MaAsLin, Coef = 1.060, *p* = 0.004, *q* = 0.038) and *Romboutsia* (*t*-test, *p* = 0.030, *q* = 0.181; MaAsLin, Coef = 0.637, *p* = 0.011, *q* = 0.068) were more abundant in male gut (Fig. 3C).

The gut microbiota tends to stabilize after three years of age^97^. In this study, we observed significant differences in gut microbiota structure among five age groups (< 20, n = 17; 20–30, n = 141; 30–40, n = 152; 40–50, n = 97; 50–60, n = 57; > 60, n = 19. Unweighted unifrac distance, Adonis, Pr (> F) = 0.006; Anosim, *p* = 0.002, Supplementary Table 3), and the higher level of *Senegalimassilia* was identified between 40 and 50 years old (t-test, 40–50 vs. > 60, *p* = 0.012,* q* = 0.393; 40–50 vs. 50–60, *p* = 0.020, *q* = 0.700; 40–50 vs. < 20, *p* = 0.003,* q* = 0.090; Supplementary Table 4). When age was considered as continuous variable in our study, five genera had a significant decreased trend as the age grew including *Bifidobacterium* (MaAsLin, Coef = − 0.053, *p* < 0.001, *q* = 0.005), *Erysipelatoclostridium* (MaAsLin, Coef = − 0.022, *p* < 0.001,* q* = 0.009), *Sellimonas* (MaAsLin, Coef = − 0.017, p = 0.002, q = 0.058), Haemophilus (MaAsLin, Coef = − 0.034, *p* = 0.005, q = 0.130) and *Butyricicoccus* (MaAsLin, Coef = − 0.023, *p* = 0.017, *q* = 0.236) (Supplementary Table 5).

The potential relationship between obesity and intestinal flora has attracted the attention of many researchers in recent years. In this study, BMI of each volunteer was calculated and included into normal weight group (n = 352), overweight group (n = 93) and underweight group (n = 38) according to relevant standards^98–100^. Nine genera were observed significantly decreased in underweight group and thirteen were decreased in overweight group (Supplementary Table 6), and after removing other confounding factors, *Oscillibacter* (*t*-test, *p* = 0.039, *q* = 0.0.545; MaAsLin, Coef = − 0.128, *p* < 0.001, *q* = 0.003) and *Holdemanella* (*t*-test, *p* = 0.016, *q* = 0.344; MaAsLin, Coef = -0.067, *p* = 0.002, *q* = 0.017) still decreased significantly as the BMI increased (Supplementary Table 7).

**Figure 4:**
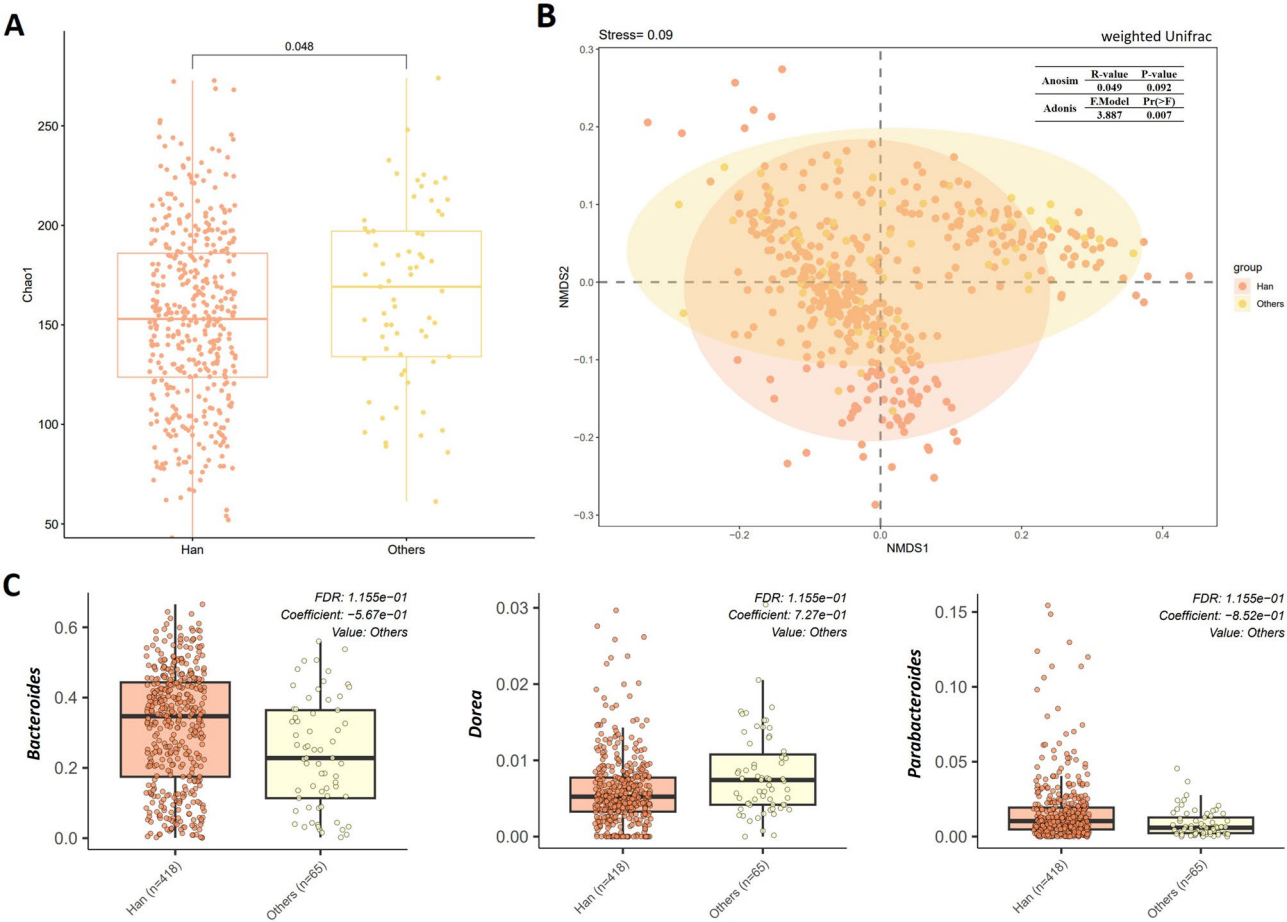
Differences in the microbiota alpha diversity, composition, and bacteria in genus-level between Han ethnic group (n = 418) and other ethnic groups (n = 65). (**A**) Chinese Han had lower richness of gut microiota than other ethnic groups. (**B**) Non-metric multidimensional scaling analysis (NMDS) based on weighted Unifrac distance matric showed the separated microbita composition between Chinese Han and others. (**C**) *Parabacteroides* and *Bacteroides* showed a higher level in Chinese Han while *Dorea* showed a lower relative abundance.

Han ethnic group counted for over 85% in this study, and the gut microbial community structure differed between Han (n = 418) and other ethnics groups (n = 65) (Chao1 index, *p* = 0.048, *q* = 0.048, K–W test, Fig. 4A; weighted unifrac distance, Adonis, Pr (> F) = 0.007, Fig. 4B). After partial out other demographic factors, living conditions and dietary habits, *Parabacteroides* (*t*-test, *p* < 0.001, *q* < 0.001; MaAsLin, Coef = -0.852, *p* = 0.004, *q* = 0.116) and *Bacteroides* (*t*-test, *p* < 0.001, *q* = 0.040; MaAsLin, Coef = − 0.567, *p* = 0.005, *q* = 0.116) were higher in Han ethnic group, and *Dorea* was lower (*t*-test, *p* = 0.004, *q* = 0.113; MaAsLin, Coef = − 0.727, *p* = 0.004, *q* = 0.116) than the other ten ethnic groups (Fig. 4C, Supplementary Tables 8, 9).

The volunteers enrolled in our study were from 62 residential areas within China (Supplementary Fig. 2), as such, habitation location could be a significant factor influencing the gut microbiota. In this study, gut microbial community structure differed between south group (n = 357) and north group (n = 126) (weighted unifrac distance, Adonis, Pr (> F) = 0.002, Supplementary Table 10).The abundance of supposedly beneficial bacteria, *Fusobacterium* (MaAsLin, Coef = 0.852, *p* = 0.015, *q* = 0.146) in the southern population of China was higher than that in the northern population, and the levels of *Bifidobacterium* (MaAsLin, Coef = − 1.472, *p* < 0.001, *q* = 0.003), *Megasphaera* (MaAsLin, Coef = − 1.205, *p* < 0.001, *q* = 0.003), and *Dialister* (MaAsLin, Coef = − 1.270, *p* = 0.006, *q* = 0.114), were higher in the northern Chinese populationafter removing other confounding factors (Supplementary Tables 11, 12).

### Different living conditions and bacteria

There are limited studies highlighting the impact of lifestyle variables on the composition and structure of the gut microbiota. In order to explore how various lifestyle variables influence the gut microbiota, we performed differential analysis of the gut microbiota in parallel with six lifestyle variables: (1) the degree of sleep deprivation, (2) state of fatigue, (3) appearance of negative emotions, (4) occurrence of oral ulcers and skin acne, and (5) smoking frequency.

**Figure 5:**
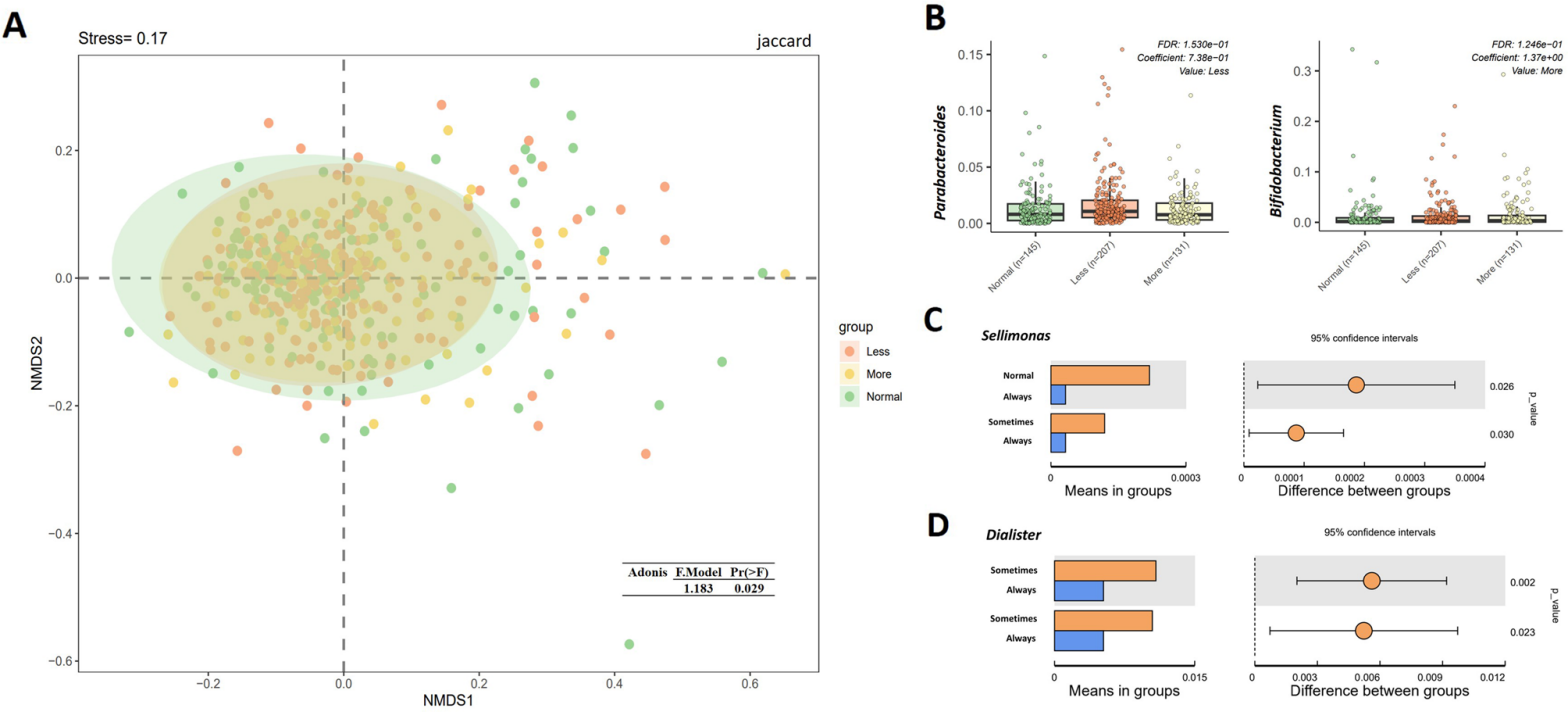
Differences in the microbiota composition and bacteria in genus-level between people different lifestyle conditions. (**A**) Gut microbiota structures among the normal sleep (n = 145), more sleep deprivation (n = 131) and less sleep deprivation (n = 207) groups showed a significant difference. (**B**) Two bacteria in genus-level were identified significantly different among three sleep deprivation groups. (**C**) T-test revealed the difference in relative abundance of *Sellimonas* in healthy Chinese volunteers with varying degrees of fatigue (normal, n = 128; sometimes, n = 283, always, n = 72). (**D**) T-test revealed the difference in relative abundance of *Dialister* in healthy Chinese volunteers with varying degrees of oral ulcers and acne (seldom, n = 246, sometimes, n = 173, always, n = 64).

According to the consensus of the American Academy of Sleep Medicine and Sleep Research Society, adults should strive for 7 h of nightly sleep while young adults require 9 h. Insufficient sleep due to occupational or recreational activities is classified as sleep deprivation^101^. One study has demonstrated a correlation between sleep deprivation and the gut dysbiosis^102^. In this study, increased sleep deprivation decreased the alpha diversity of the gut microbiome (Supplementary Table 13)although the differences were not significant, and the gut microbiota structures among the normal sleep (n = 145), more sleep deprivation (n = 131) and less sleep deprivation (n = 207) groups showed a significant difference (Binary-jaccard distance, Adonis, Pr (> F) = 0.029, Fig. 5A) The abundance of *Bifidobacterium* in the normal sleep group was significantly lower than that in the more sleep deprived (MaAsLin, Coef = 1.367, *p* = 0.001, *q* = 0.125) groups, while *Parabacteroides* showed a significant higher relative abundance in less sleep group than that in normal sleep MaAsLin, Coef = 0.738, *p* = 0.002, *q* = 0.153, Fig. 5B, Supplementary Tables 14, 15).

Fatigue is a prevalent symptom frequently encountered in daily life. It can be classified into two types: acute and chronic fatigue. Acute fatigue usually resolves after rest or treatment of the underlying condition, while chronic fatigue is an enduring debilitating process. Therefore, they differ at least in terms of frequency of experiencing fatigue. Our results showed that as the fatigue level and frequency increased (normal, n = 128; sometimes, n = 283, always, n = 72), the level of *Sellimonas* decreased gradually (*t*-test, normal vs. always, *p* = 0.026, *q* = 0.731; sometimes vs. always, *p* = 0.030, *q* = 0.766 (Fig. 5C, Supplementary Table 16).

Previous studies have shown that the intestinal microbiome plays a role in stress response, inflammation, depression and anxiety, but specific changes in microbial composition and structure were not clear^53–55^. According to the survey conducted in 2021, the most negative emotions experienced by Chinese individuals were anxiety, anger, fear and sadness^103^.We identified five genera which were significant different in relative abundance, and *Negativibacillus* (*t*-test, *p* = 0.001, *q* = 0.153; MaAsLin, Coef = -0.687, *p* < 0.001, *q* = 0.037) showed a significant higher level after removing other confounding factors (Supplementary Tables 17, 18). Taken together, our results showed that sleep deprivation, the fatigue level, and negative emotions had associations with the gut microbiota community structure.

Some unhealthy lifestyle factors may lead to the development of oral ulcers and acne. The underlying mechanism is likely related to proinflammatory response^6, 7, 56^. Probiotic therapy has been shown to modulate inflammation and improve symptoms of both oral ulcers and acne^2–5, 8, 9, 57–60^. Therefore, it is necessary to characterize the gut microbiome in this population. Our results showed that as the frequency of oral ulcers and acne increased, the abundance of supposedly beneficial bacteria, *Dialister* significantly decreased gradually although the decrease was not significant after removing the confounding factors (seldom, n = 246, sometimes, n = 173, always, n = 64; *t*-test, seldom vs. always, *p* = 0.002, *q* = 0.199; sometimes vs. always, *p* = 0.023, *q* = 0.527, Fig. 5D, Supplementary Table 19).

A previous study showed that nicotine intake can lead to intestinal flora imbalance in mice^61^. The effects of cigarette smoking on intestinal disorders include changes in intestinal irrigation and the gut microbiome, increases in the permeability of the mucosa, and impaired mucosal immune responses^62, 63^. Cigarette smoke may influence the gut microbiota by increasing the pH value of the intestinal tract, which could be conducive to the growth of some bacteria, leading to the imbalance of the intestinal flora structure^64, 65^. Among the smoking-related phenotypes (never, n = 387; sometimes, n = 44; always, n = 52), we observed significant differences in gut microbiota structure (Binary-jaccard distance, Adonis, Pr (> F) = 0.029; Anosim, *p* = 0.012, Supplementary Table 20). An increase in smoking frequency decreased the richness and evenness of the gut microbiome (Chao1 index, *p* = 0.007, *q* = 0.021; Shannon index, *p* = 0.037, *q* = 0.112, K–W test, Supplementary Table 21), and the relative abundance of *Gastranaerophilales* (*t*-test, never vs. sometimes, *p* < 0.001, *q* = 0.008; never vs. always, *p* = 0.048, *q* = 0.217), *Catenibacterium* (*t*-test, never vs. sometimes, *p* < 0.001, *q* = 0.017; never vs. always, *p* < 0.001, *q* = 0.013)*,* and *Coprobacter* (*t*-test, never vs. sometimes, *p* = 0.013, *q* = 0.134; never vs. always, *p* = 0.041, *q* = 0.194) (Supplementary Table 22). 

### Different dietary habits and bacteria

Dietary habits have been suggested to be intimately related to the gut microbiome^20, 66–68^. In order to explore the impact of different dietary habits on the composition of the intestinal flora, we analyzed the impact of three dietary preferences, (1) starch intake (cereal, rice, flour-based foods, and high-starch foods), (2) protein intake (bean products, dairy, eggs, and meat), and (3) dietary preference (meat-heavy diet, vegetable-heavy diet, or a balanced diet of meat and vegetables), on the structure of the gut microbiota in the healthy Chinese volunteers.

**Figure 6:**
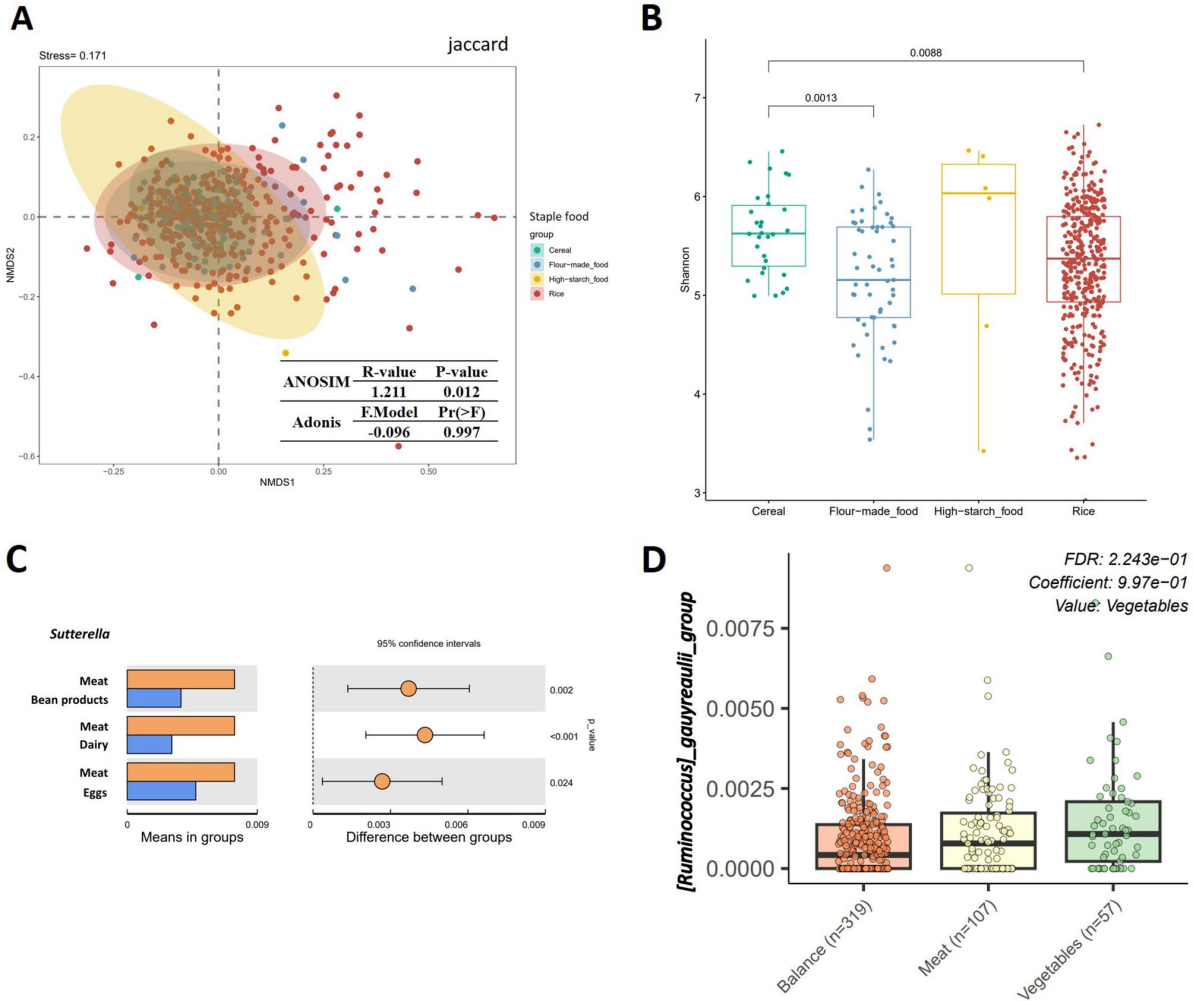
Differences in the microbiota alpha diversity, composition, and bacteria in genus-level between people with different dietary habits. (**A**) Gut microbiota structures among the different starch intake groups (cereal, n = 30; rice, n = 390; flour-based, n = 57; high-starch, n = 6) showed a significant difference. (**B**) Healthy Chinese who took cereal as their staple food had higher level of gut microbita diversity than who consumed flour-based foods or rice. (**C**) T-test revealed the difference in relative abundance of *Sutterellas* in healthy Chinese volunteers with different types of protein intake (bean products, n = 47; dairy, n = 68; eggs, n = 95; meat, n = 273). (**D**) Healthy Chinese who preferred vegetable diet (n = 57) had higher level of *[Ruminococcus]_gnavus_group* than who preferred balanced (n = 319) or meat (n = 107) diet.

In order to assess how various types of starch affect the healthy Chinese gut microbiota, the volunteers were grouped according to the predominant starch type consumed (cereal, n = 30; rice, n = 390; flour-based, n = 57; high-starch, n = 6), and microbiota was analyzed. We found that healthy Chinese volunteers who intake different starch had a significant different gut microbiota structure (Binary-jaccard distance, Adonis, Pr (> F) = 0.024, Fig. 6A, Supplementary Table 23). Compared with volunteers who consumed rice or flour-based foods as their staple food, the volunteers consuming cereal had a higher gut microbiota community diversity (Shannon index, cereal vs. flour-based, *p* = 0.001, *q* = 0.008; cereal vs. rice, *p* = 0.009, *q* = 0.026, K–W test, Fig. 6B, Supplementary Table 24). High-starch consumption was associated with lower relative abundance of *Megamonas* (*t*-test, high-starch vs. flour-based, *p* < 0.001, *q* = 0.029; high-starch vs. cereal, *p* = 0.001, *q* = 0.168; high-starch vs. rice, *p* < 0.001, *q* < 0.001) and *Bilophila* (*t*-test, high-starch vs. flour-based, *p* = 0.010, *q* = 0.355; high-starch vs. cereal, *p* = 0.026, *q* = 0.690; high-starch vs. rice, *p* < 0.001, *q* = 0.011), and rice consumption was associated with a higher level of Parabacteroides (*t*-test, rice vs. flour-based, *p* = 0.009, *q* = 0.147; rice vs. cereal, *p* = 0.045, *q* = 0.343) compared with rice or flour-based foods consumption although these associations were not significant after removing the other confounding factors (Supplementary Table 25).

We then grouped the fecal stool samples according to the predominant protein source consumed by the volunteers (bean products, n = 47; dairy, n = 68; eggs, n = 95; meat, n = 273), and found that the level of *Sutterella* (*t*-test, meat vs. bean products, *p* = 0.002, *q* = 0.116; meat vs. dairy, *p* < 0.001, *q* = 0.033; meat vs. eggs, *p* = 0.024, *q* = 0.677) and *Mitsuokella* (*t*-test, meat vs. bean products, *p* = 0.011, *q* = 0.230; meat vs. dairy, *p* = 0.003, *q* = 0.193) were significantly higher in people who consumed meat as their primary protein source compared to people to who consumed other primary protein source. The abundance of *Fusobacterium* was significantly lower in people who consumed dairy as their primary protein source compared to people who derived the majority of their protein from meat or eggs (*t*-test, dairy vs. meat, *p* < 0.001, *q* = 0.033; dairy vs. eggs, *p* = 0.028, *q* = 0.750) although the different levels were not significant after removing the confounders (Fig. 6C, Supplementary Table 26). These results demonstrate that the dietary protein source was associated with the community structure of the intestinal flora.

Next, we were interested in determining how general dietary preference was associated with the gut microbiome of a healthy Chinese population. The stool samples were divided into three groups based on the dietary preference of a meat diet (n = 107), vegetable diet (n = 57), or a balanced diet of both meat and vegetables (n = 319). Several bacteria in genus-level were identified between any two groups, but only *[Ruminococcus]_gnavus_group* showed a significant higher relative abundance in vegetable-diet preference volunteers compared with who preferred balanced diet after removing the confounding factors(*t*-test, *p* = 0.046, *q* = 0.526; MaAsLin, Coef = 0.977, *p* = 0.001, *q* = 0.224) (Fig. 6D, Supplementary Table 27).

## Discussion

Numerous studies have shown that several factors, including diet and lifestyle variables, influence the diversity, structure, and composition of the gut microbiota^16–22, 69^. Delineating the composition of the gut microbiome in populations with various genetic backgrounds and lifestyle patterns may be beneficial in understanding the mechanisms linking lifestyle patterns and overall health and disease risk. Here, we collected questionnaire data and fecal stool samples from 483 healthy Chinese volunteers that spanned 11 ethnic groups and were from 62 residential areas. We then performed 16S rRNA sequencing on the microbial DNA isolated from the stool samples to assess the composition of the gut microbiota in relation to lifestyle and dietary variables.

Our results showed that *Bacteroides* and Faecalibacterium were the most abundant genera in the healthy Chinese gut microbiome, which is consistent with a previous study that assessed the gut microbiota in healthy Chinese volunteers^35^. This result is consistent with population-level studies conducted in African populations, but it differs from studies conducted on European and American populations, in which *Bacteroides* and *Firmicutes* were the primary enterotypes^18, 70^. As reported in previous studies^26, 69^, *Prevotella*- and *Bacteroides*-rich compositions were found to be relatively non-overlapping in energy obtain. People with a *Bacteroides*–rich enterotype obtain energy from carbohydrates and proteins, while people with a *Prevotella*-rich enterotype mainly degrade mucin glycoproteins existing in the intestinal mucosal layer.

*Bacteroides* is an important and abundant member of the gut microbiome and is a core microorganism of common enterotypes^71^. In this study, we found that *Bacteroides* levels increased in Han ethnic group. People who consumed eggs as their main protein source, and always occurred to oral ulcers and skin acne had an increased relative abundance of *Bacteroides*, which is consistent with a previous study^75^, although the significance disappeared after the confounder was removed. In previous studies, *Bacteroides* was reported to degrade glycans and proteins as nutritional sources^43^, and *Bacteroides* levels have been shown to be closely related to dietary habits^72^. Adjusting *Bacteroides* levels by modifying the dietary structure may be a way to improve sub-health lifestyle patterns. Additionally, it was reported that the nutrients obtained by *Bacteroides* via glycan degradation can be used as an energy source by other microorganisms, and the content of *Bacteroides* in the gut is related to other microorganisms^73^. In addition, *Bacteroides* has been reported to be related to the immune system^74^, and as such, has been shown to activate CD4^+^ cells by producing zwitterionic polysaccharide (ZPS), which triggers the immune system..

*Prevotella*, similar to *Bacteroides*, is a driving taxon in bacterial enterotypes of the gut microbiome. Hydrolases expressed by *Prevotella* are essential to the degradation of plant fibers^76^, which supports previous studies that have shown that *Prevotella* is enriched in populations that consume a non-Western diet and/or fiber-rich diet^18, 21, 77^. Our results showed that *Prevotella* abundance was higher in healthy Chinese male volunteers, and the increased level of *Prevotella* in gut was accompanied by elevated BMI. Additionally, *Coprococcus*, a butyrate-producing bacterium involved in dopamine-related biological pathways^78^, was found to be significantly lower in volunteers who had higher BMI index, which was similar to *Prevotella*.

Upon further analysis, we speculated that different living habits might affect the structure and composition of the gut microbiome in a couple of ways. First, some living habits, such as sleep deprivation, can increase stress and gut permeability, which may lead to abdominal distension, stomachache, and inflammation—all of which can reduce the diversity of the gut microbiome^79–81^. Second, intestinal inflammation will continue to erode the protective mucosa that protects the intestinal lining from bacterial invasion. Since the stomach wall is thin, microbial by-products, and possibly even entire bacteria, may pass through the stomach wall, triggering an inflammatory reaction of the immune system, thus forming a vicious circle. Through this process, the inflammatory process may induce fatigue^82, 83^. From our results, we found that the four staple foods were associated with the growth of different kinds of bacteria. Therefore, it can be speculated that a reasonable mode of carbon and water intake is to eat an appropriate amount of high-starch food and to choose a variety of staple foods.

There are some limitations in our study. First, although we collected demographic, lifestyle, and dietary information from all of the volunteers, our study is limited by the lack of comprehensive physiological indices and detailed lifestyle and dietary information. Such information is imperative for fully interpreting the data. Furthermore, compared with a previous study^73^ that examined 150 host phenotypic features, our research is still not detailed enough in regards to phenotypic diversity. Second, while we determined the differential abundance of microorganisms in relation to various phenotypes, we did not investigate differences in metabolic pathways or metabolites. As such, we were not able to reveal any information regarding the metagenome of the volunteers. Nonetheless, the microbial profiles acquired in this study do help elucidate the gut microbiota in the healthy Chinese population at baseline. Third, the correlations established in this study cannot determine causal relationships between the gut microbiota and lifestyle variables. In order to determine causal relationships, animal experiments and in vitro experiments, as well as intervention experiments, need to be performed. Fourth, the data presented in this study are from 483 healthy Chinese volunteers. However, while our total sample number is one of the largest reported in the literature, our sample number per variable is small, and additional samples are needed to verify the statistical analysis. In a follow-up study, we hope to further accumulate relevant data to improve the dimensional phenotypic analyses. There is no doubt that changing the structure and composition of intestinal microorganisms may become an important part of precision medicine in the twenty-first century.

In summary, our study compared the gut microbiota with demographic, lifestyle, and dietary variables in healthy Chinese volunteers. We found that the most abundant genera in the healthy Chinese gut microbiome were *Prevotella* and *Bacteroides.* Additionally, nine clinical and questionnaire-based phenotype covariates were found to be associated with the composition of the gut microbiota. The results of this study provide a foundation for elucidating the gut microbiome in the Chinese population at baseline. Moreover, understanding the complex interactions between the gut microbiome and various lifestyle and dietary variables prior to disease onset may help prevent disease or guide disease treatment.

## Materials and methods

### Study population and research data

After excluding the volunteers who were diagnosed with any illness, we recruited 483 healthy volunteers ranging in age from 5 to 80 years old. Information was collected from each recruited volunteer via an online questionnaire based on their lifestyles over the past week. The questionnaire contained 28 questions grounded on contemporary research that elucidates the factors associated with the gut microbiome—7 of which regarded basic information, such as age and gender, and the remaining 21 were questions relating to dietary and lifestyle variables divided into three categories: 1) bowel habits (four questions), dietary habits (five questions), allergens and health (five questions), and other lifestyle habits (seven questions). Detailed information regarding the questionnaire questions is listed in Table 1.

To gain a better understanding of the gut microbiome features among individuals with different lifestyles in China, the initial step involves characterizing distinct groups. According to WHO guidelines, adults should engage in 150–300 min of exercise per week^104^. Participants were classified into three groups based on self-reported exercise frequency: “No” indicated minimal physical activity, “1–2 times a week” indicated insufficient exercise, and “3 or more times” indicated compliance with guidelines. Fatigue was categorized into three groups based on responses to the question “Have you experienced recent feelings of tiredness?”: “No” indicated sustained vitality, “Sometimes” indicated recoverable fatigue, and “Always” indicated persistent weariness^105^. Personal alcohol consumption habits were categorized as “Never,” “Seldom,” (occasional consumption of alcohol in social settings) or “Often” (daily alcohol intake). Smoking frequency was classified as “No,” “Seldom” (< 15 cigarettes/day), or “Often” (≥ 15 cigarettes/day), with insight provided by previous studies on tobacco dependence^106, 107^. Sleep deprivation was categorized as “No,” “Seldom” (1–2 days/week), or “Often” (≥ 3 days/week) based on self-reported insufficient sleep (< 7 h/night)^108^. Participants’ mysophobia was classified as “No” or “Yes,” while negative emotions were determined by recent experiences of anxiety, anger, fear, or sadness^89^.

This study was performed with the approval of the Ethical Committees of Beijing Institute of Microbiology and Epidemiology, and written informed consent from all of the volunteers was obtained. The methods were all carried out in accordance with the approved guidelines.

### Sample collection and DNA extraction

Approximately 5 mL of feces for each volunteer was collected using sterile fecal sampling tubes (SARSTEDT AG & Co. KG, Nümbrecht, Germany) to lower the risk of bias. All samples were stored at − 80 °C prior to isolating genomic DNA using the TIANamp Stool DNA Kit (Tiangen Angen Biotech (Beijing) Co., Ltd., Beijing, China) following the manufacturer’s instructions. DNA integrity was evaluated by agarose gel electrophoresis on a 1.2% agarose gel with 1 × TAE Buffer running at a constant voltage of 110 V. A Qubit R3.0 Fluorometer (Thermo Fisher Scientific Inc., Waltham, Massachusetts, USA) was used to assess the quality of the DNA and to measure DNA purity and concentration. DNA with an OD260/OD280 ratio between 1.8 and 2.0 was considered pure, and all of the DNA concentrations were higher than 2.5 ng/μL.

### 16S rRNA gene sequencing and bioinformatics analyses

Of the 1078 volunteers recruited, 16S rRNA sequencing was performed on 856 volunteers. One-step PCR was used to prepare the PCR Illumina sequencing libraries in a 25-uL reaction containing template DNA (25 ng), forward and reverse primers for the V3–V4 region (333 nmol each), and KAPA Hi-Fi PCR master mix (Kapa Biosystems, Boston, MA, USA). The forward and reverse primers used to amplify the V3–V4 region were as follows: forward primer: 5’-CCTAYGGGRBGCASCAG-3’ and reverse primer: 5’-GGACTACNNGGGTATCTAAT-3’. The PCR conditions were as follows: enzyme activation step at 95 °C for 3 min, followed by 20 cycles of 15 s at 98 °C, 30 s at 50 °C, 40 s at 72 °C, and 10 min at 72 °C, with a final hold at 10 °C. The cDNA was purified by the addition of Clean Beads (Beckman Coulter Inc., Brea, California, USA) and then sequenced on an Illumina HiSeq2500 platform (Illumina, Inc., San Diego, California, USA), which generated approximately 4.5 million reads of 16S rRNA V3–V4 amplicons comprising the partial C3 region (341F, 17 bp), full V3 region (57 bp), full V4 region (62 bp), and partial C5 region (806R, 20 bp). Samples that lacked volunteer data, from a patient that had an illness that may have biased the results, or inadequate sequencing data were excluded from the analysis. A total of 483 samples remained, all of which were included in the analysis.

### Raw data filtering, classification, and annotation

The adaptors and PCR primers were removed from the reads, and these paired-end reads were denoised, filtered and joined using the DADA2 software package^84^ implemented in QIIME 2^85^, and the number of reads from each sample was rarefied to 2000. Rare amplicon sequence variants (ASVs) with total frequency less than 5, or was observed in one sample, or relative abundance was below 0.1% were removed. Taxonomy was assigned to ASV using the feature-classifier, a classify-sklearn naive Bayes taxonomy classifier using machine learning against the Silva 138 database^86^.

### Diversity analysis and variation analysis

The QIIME2 diversity alpha plugin produced alpha diversity measures (Chao1 richness index and Shannon diversity index), which were used to analyze the alpha diversity level of different groups. The differences of alpha diversity was further tested using Kruskal–Wallis analysis (K–W test). The unweighted Unifrac, weighted Unifrac, Bray–curtis and Binary-jaccard distance matrices between samples were used for non-metric multidimensional scaling analysis (NMDS) at the ASV level^87, 88^, and adonis and anosim analysis were used to assess the explanatory power of grouping factors on sample dissimilarities.

In order to discover biomarkers with statistical differences, we also applied an inter-group *t* test (figures were generated with DRAW package for Perl). Multivariate analysis by linear models (MaAsLin 2 R package, version 1.7.3)^92^ was further used to remove the confounding factors and identify bacterial genera associated with each factor. When identifying the biomarkers within one factor groups, the other factors were considered to be confounding factors. Only those taxa that were present in more than 10% of samples, and whose relative abundances were > 0.01% were included. Benjamini–Hochberg method was used to adjust *P*-values, given as *Q*-values, and the α level was set at 0.05 (two-sided) throughout all tests.

### Methods of bacterial flora classification

We used two methods of bacterial flora classification in this study. The first method was based on the detection rate (sample size of a bacterium detected/total sample size). The second method was based on cluster analysis^26^.

In this study, cluster analysis was achieved via R language. We used the partitioning around the medoids (PAM) algorithm^7^, which supports any arbitrary distance measure to cluster the abundance profiles. Here, a probability distribution distance metric^8^ related to Jensen-Shannon divergence (JSD) was applied. The distance $D\left(a, b\right)$ between samples *a* and *b* is defined as

$$D\left(a, b\right)=\sqrt{\frac{1}{2}KLD\left({p}_{a}, \frac{{p}_{a}+{p}_{b}}{2}\right)+ \frac{1}{2}KLD\left({p}_{b}, \frac{{p}_{a}+{p}_{b}}{2}\right)}$$

where ${p}_{a}$ and ${p}_{b}$ are the abundance distributions of samples *a* and *b*, and $KLD\left(x,y\right)$ is the Kullback–Leibler divergence between *x* and *y*, defined as

$$KLD\left(x,y\right)=\sum_{i}{x}_{i}\mathrm{log}\frac{{x}_{i}}{{y}_{i}}$$

A pseudocount of 0.000001 was applied to the abundance distributions to avoid zero in the equation. Then, using the JSD, we assessed the Calinski–Harabasz (CH) index to evaluate the optimum number of clusters^9^; it is defined as

$${CH}_{k}=\frac{\frac{{B}_{k}}{k-1}}{\frac{{W}_{k}}{n-k}}$$

where ${B}_{k}$ is the squared distance between all points *i* and *j*, for which *i* and *j* are not in the same cluster, and ${W}_{k}$ is the squared distance between all points *i* and *j*, for which *i* and *j* are in the same cluster. The result indicated that the CH index reached the maximum when divided into two clusters, so the number of clusters was set to *k* = 2.

Contingency tables were used for checking the independence between enterotypes and the other phenotypes, and the results showed that the distribution of enterotypes was not affected by demographic characteristics and phenotypic information (for each phenotype *p* > 0.05).

Here, we chose the top 35 bacteria in genus-level in abundance to cluster. Last, the clustering quality was assessed using the silhouette validation technique. Values derived from the silhouette are located between − 1 and + 1, and the value in this research was 0.262. In theory, the smaller the gap between the acquired value and the value derived from the silhouette, the more accurate the clustering technique.

### Supplementary Information

Supplementary Information.

## Data Availability

The raw sequence data reported in this paper have been deposited in the Genome Sequence Archive^90^ in National Genomics Data Center^91^, China National Center for Bioinformation/Beijing Institute of Genomics, Chinese Academy of Sciences (GSA: CRA009598) that are publicly accessible at https://ngdc.cncb.ac.cn/gsa/s/87n639Hu.
