# Supplementary material for: Lifestyle patterns influence the composition of the gut microbiome in a healthy Chinese population
Source: Sci Rep. 2023 Sep 2;13:14425. doi: 10.1038/s41598-023-41532-4 (PMC10475076; doi:10.1038/s41598-023-41532-4)
Supplement: Supplementary file 1 — Supplementary Information. [file 41598_2023_41532_MOESM1_ESM.docx]

**Supplementary Table 1. Associations of microbiota with enterotype at genus level adjusting for demographic factors including ethnicity, age, gender, ethnicity, BMI and habitation, living conditions including state of fatigue, degree of sleep deprivation, appearance of negative emotions, occurrence of oral ulcers and skin acne, and smoking frequency, and dietary habits including starch intake, protein intake and dietary preference using MaAsLin analysis**

| **Feature** | **Group** | **Coefficient** | ***P*-value** | ***Q*-value** |
| --- | --- | --- | --- | --- |
| g_Prevotella | Type2 | 8.736 | <0.001 | <0.001 |
| g_Bacteroides | Type2 | -2.148 | <0.001 | <0.001 |
| g_Alloprevotella | Type2 | 2.250 | <0.001 | <0.001 |
| g_Holdemanella | Type2 | 1.686 | <0.001 | <0.001 |
| g_Eggerthella | Type2 | -1.212 | <0.001 | <0.001 |
| g_[Ruminococcus]_gnavus_group | Type2 | -2.315 | <0.001 | <0.001 |
| g_[Eubacterium]_ruminantium_group | Type2 | 2.516 | <0.001 | <0.001 |
| g_Flavonifractor | Type2 | -1.598 | <0.001 | <0.001 |
| g_Tyzzerella | Type2 | -2.170 | <0.001 | <0.001 |
| g_Collinsella | Type2 | 1.837 | <0.001 | <0.001 |
| g_NK4A214_group | Type2 | 1.666 | <0.001 | <0.001 |
| g_[Eubacterium]_coprostanoligenes_group | Type2 | 1.791 | <0.001 | <0.001 |
| g_Blautia | Type2 | -0.530 | <0.001 | <0.001 |
| g_Family_XIII_UCG-001 | Type2 | 0.669 | <0.001 | <0.001 |
| g_UCG-002 | Type2 | 1.603 | <0.001 | <0.001 |
| g_UCG-010 | Type2 | 1.019 | <0.001 | <0.001 |
| g_[Ruminococcus]_gauvreauii_group | Type2 | 1.007 | <0.001 | <0.001 |
| g_Clostridia_vadinBB60_group | Type2 | 0.879 | <0.001 | <0.001 |
| g_Anaerostipes | Type2 | -0.800 | <0.001 | <0.001 |
| g_UCG-005 | Type2 | 1.548 | <0.001 | <0.001 |
| g_Hungatella | Type2 | -0.621 | <0.001 | <0.001 |
| g_Bifidobacterium | Type2 | -1.538 | <0.001 | <0.001 |
| g_Coprococcus | Type2 | 1.168 | <0.001 | <0.001 |
| g_Erysipelatoclostridium | Type2 | -0.700 | <0.001 | <0.001 |
| g_Parabacteroides | Type2 | -0.963 | <0.001 | <0.001 |
| g_Fusicatenibacter | Type2 | -0.710 | <0.001 | <0.001 |
| g_Intestinibacter | Type2 | -1.123 | <0.001 | <0.001 |
| g_Lachnospiraceae_NK4A136_group | Type2 | 1.140 | <0.001 | <0.001 |
| g_Sellimonas | Type2 | -0.577 | <0.001 | <0.001 |
| g_Muribaculaceae | Type2 | 1.333 | <0.001 | <0.001 |
| g_Dialister | Type2 | 1.742 | <0.001 | <0.001 |
| g_Desulfovibrio | Type2 | 0.920 | <0.001 | <0.001 |
| g_Lachnospiraceae_UCG-001 | Type2 | -0.936 | <0.001 | <0.001 |
| g_Paraprevotella | Type2 | 1.529 | <0.001 | <0.001 |
| g_Adlercreutzia | Type2 | -0.745 | <0.001 | <0.001 |
| g_Sutterella | Type2 | 1.546 | <0.001 | <0.001 |
| g_Dorea | Type2 | 0.685 | <0.001 | <0.001 |
| g_GCA-900066575 | Type2 | 0.563 | <0.001 | 0.001 |
| g_Allisonella | Type2 | 0.634 | <0.001 | 0.001 |
| g_[Eubacterium]_xylanophilum_group | Type2 | -0.774 | <0.001 | 0.002 |
| g_Lactobacillus | Type2 | 0.772 | 0.001 | 0.003 |
| g_UBA1819 | Type2 | -0.738 | 0.002 | 0.004 |
| g_Escherichia-Shigella | Type2 | -1.054 | 0.002 | 0.005 |
| g_UCG-003 | Type2 | 0.739 | 0.003 | 0.006 |
| g_Monoglobus | Type2 | -0.823 | 0.003 | 0.007 |
| g_Subdoligranulum | Type2 | 0.786 | 0.005 | 0.010 |
| g_Clostridia_UCG-014 | Type2 | 1.198 | 0.005 | 0.010 |
| g_Christensenellaceae_R-7_group | Type2 | 1.089 | 0.005 | 0.010 |
| g_Megamonas | Type2 | 1.322 | 0.005 | 0.011 |
| g_Senegalimassilia | Type2 | 0.326 | 0.008 | 0.015 |
| g_Lachnospiraceae_FCS020_group | Type2 | 0.464 | 0.012 | 0.022 |
| g_Alistipes | Type2 | -0.807 | 0.012 | 0.022 |
| g_Lachnospiraceae_UCG-010 | Type2 | 0.625 | 0.013 | 0.025 |
| g_[Eubacterium]_ventriosum_group | Type2 | -0.596 | 0.015 | 0.026 |
| g_Veillonella | Type2 | -0.627 | 0.018 | 0.030 |
| g_Holdemania | Type2 | -0.354 | 0.017 | 0.030 |
| g_Bilophila | Type2 | -0.616 | 0.018 | 0.030 |
| g_Parasutterella | Type2 | -0.854 | 0.023 | 0.039 |
| g_Lachnospira | Type2 | -0.510 | 0.034 | 0.055 |
| g_Lachnospiraceae_UCG-003 | Type2 | -0.484 | 0.034 | 0.055 |
| g_Lachnospiraceae_UCG-004 | Type2 | 0.423 | 0.041 | 0.066 |
| g_Oscillibacter | Type2 | -0.459 | 0.047 | 0.073 |
| g_Erysipelotrichaceae_UCG-003 | Type2 | -0.573 | 0.066 | 0.102 |
| g_Negativibacillus | Type2 | 0.331 | 0.117 | 0.178 |
| g_Faecalibacterium | Type2 | 0.284 | 0.140 | 0.209 |
| g_[Ruminococcus]_torques_group | Type2 | 0.300 | 0.163 | 0.238 |
| g_Incertae_Sedis | Type2 | -0.330 | 0.167 | 0.238 |
| g_Haemophilus | Type2 | -0.455 | 0.166 | 0.238 |
| g_Enterococcus | Type2 | -0.237 | 0.174 | 0.241 |
| g_Agathobacter | Type2 | 0.393 | 0.173 | 0.241 |

MaAsLin, multivariate association with linear models.

**Supplementary Table 2. Associations of microbiota with gender at genus level using t-test**

| **Feature** | **Average Relative Abundance** | | ***P*-value** | ***Q*-value** |
| --- | --- | --- | --- | --- |
|  | **Female** | **Male** |  |  |
| g_Lachnospiraceae_NK4A136_group | 0.0123 | 0.0058 | <0.001 | <0.001 |
| g_GCA-900066575 | 0.0003 | 0.0001 | <0.001 | <0.001 |
| g_Lachnospiraceae_ND3007_group | 0.0024 | 0.0013 | <0.001 | 0.001 |
| g_[Eubacterium]_xylanophilum_group | 0.0005 | 0.0002 | <0.001 | 0.015 |
| g_UCG-010 | 0.0006 | 0.0002 | <0.001 | 0.021 |
| g_[Eubacterium]_siraeum_group | 0.0024 | 0.0009 | <0.001 | 0.028 |
| g_Lachnospiraceae_UCG-001 | 0.0014 | 0.0008 | 0.002 | 0.051 |
| g_Family_XIII_UCG-001 | 7.12E-05 | 3.23E-05 | 0.003 | 0.066 |
| g_Subdoligranulum | 0.0171 | 0.0133 | 0.004 | 0.066 |
| g_Eisenbergiella | 9.32E-05 | 1.08E-05 | 0.004 | 0.066 |
| g_Gastranaerophilales | 0.0002 | 3.98E-05 | 0.005 | 0.066 |
| g_Lachnospiraceae_UCG-004 | 0.0049 | 0.0038 | 0.005 | 0.066 |
| g_Prevotellaceae_NK3B31_group | 0.0021 | 0.0002 | 0.005 | 0.071 |
| g_[Eubacterium]_eligens_group | 0.0084 | 0.0054 | 0.006 | 0.072 |
| g_Clostridia_UCG-014 | 0.0062 | 0.0033 | 0.008 | 0.087 |
| g_UCG-003 | 0.0014 | 0.0010 | 0.010 | 0.103 |
| g_Faecalibacterium | 0.1024 | 0.0874 | 0.011 | 0.106 |
| g_Lachnospiraceae_UCG-010 | 0.0012 | 0.0009 | 0.0120 | 0.106 |
| g_Colidextribacter | 0.0008 | 0.0006 | 0.013 | 0.106 |
| g_Marvinbryantia | 7.86E-05 | 3.25E-05 | 0.013 | 0.106 |
| g_Muribaculaceae | 0.0030 | 0.0013 | 0.013 | 0.106 |
| o_Rhodospirillales;f_uncultured;g_uncultured | 0.0028 | 0.0012 | 0.014 | 0.107 |
| g_Akkermansia | 0.0032 | 0.0003 | 0.014 | 0.107 |
| g_Butyricicoccus | 0.0048 | 0.0040 | 0.024 | 0.174 |
| f_Oscillospiraceae;g_uncultured | 0.0006 | 0.0004 | 0.026 | 0.175 |
| g_Coprobacter | 0.0004 | 0.0002 | 0.027 | 0.181 |
| g_UCG-002 | 0.0072 | 0.0054 | 0.029 | 0.181 |
| g_Romboutsia | 0.0025 | 0.0039 | 0.030 | 0.181 |
| g_Fusobacterium | 0.0037 | 0.0111 | 0.031 | 0.181 |
| g_Barnesiella | 0.0023 | 0.0015 | 0.034 | 0.194 |
| g_Haemophilus | 0.0016 | 0.0008 | 0.041 | 0.224 |
| g_Fenollaria | 1.66E-05 | 3.34E-06 | 0.04 | 0.230 |
| g_Collinsella | 0.0055 | 0.0096 | 0.045 | 0.235 |
| g_UBA1819 | 0.0005 | 0.0002 | 0.048 | 0.242 |

**Supplementary Table 3. Statistical test results of inter-group and intra-group differences among age groups using Adonis and Anosim analysis and four distance matrices were applied: Unweighted Unifrac, Weighted Unifrac, Bray-curtis and Binary-jaccard**

| **Distance Matrices** | **Adonis** | | **Anosim** | |
| --- | --- | --- | --- | --- |
|  | **F. Model** | **Pr (> F)** | **R-value** | ***p*-value** |
| **Unweighted Unifrac** | 1.5148 | 0.006 | 0.0402 | 0.0002 |
| **Weighted Unifrac** | 2.3150 | 0.004 | 0.0114 | 0.179 |
| **Bray-curtis** | 1.4889 | 0.001 | 0.0422 | 0.001 |
| **Binary-jaccard** | 1.2950 | 0.001 | 0.0360 | 0.015 |

**Supplementary Table 4. Associations of microbiota with age at genus level using t-test**

| **Feature** | **Average Relative Abundance** | | ***P*-value** | ***Q*-value** |
| --- | --- | --- | --- | --- |
|  | **40-50** | **>60** |  |  |
| g_Sutterella | 0.0054 | 0.0008 | <0.001 | 0.006 |
| g_Allisonella | 9.69E-05 | 0.0000 | <0.001 | 0.072 |
| g_Collinsella | 0.0063 | 0.0026 | 0.009 | 0.368 |
| g_Negativibacillus | 0.0003 | 9.89E-06 | 0.009 | 0.368 |
| g_Senegalimassilia | 0.0001 | 1.18E-05 | 0.012 | 0.393 |
| g_Fusobacterium | 0.0052 | 0.0002 | 0.016 | 0.422 |
| g_Lachnospiraceae_UCG-010 | 0.0011 | 0.0005 | 0.024 | 0.513 |
| g_Family_XIII_UCG-001 | 5.58E-05 | 1.47E-05 | 0.030 | 0.513 |
| g_Lachnoclostridium | 0.0123 | 0.0072 | 0.032 | 0.513 |
| g_Tyzzerella | 0.0032 | 0.0012 | 0.032 | 0.513 |
| g_[Clostridium]_methylpentosum_group | 4.97E-05 | 0.0000 | 0.038 | 0.548 |
| g_Gastranaerophilales | 0.0002 | 1.6E-05 | 0.043 | 0.561 |
| **Feature** | **Average Relative Abundance** | | ***P*-value** | ***Q*-value** |
|  | **40-50** | **50-60** |  |  |
| g_Lachnospiraceae_FCS020_group | 0.0004 | 0.0002 | 0.009 | 0.700 |
| g_Senegalimassilia | 0.0001 | 1.65E-05 | 0.020 | 0.700 |
| g_Haemophilus | 0.0013 | 0.0004 | 0.028 | 0.700 |
| f_Lachnospiraceae;g_uncultured | 0.0002 | 8.54E-05 | 0.048 | 0.700 |
| o_Rhodospirillales;f_uncultured;g_uncultured | 0.0012 | 0.0045 | 0.049 | 0.700 |
| **Feature** | **Average Relative Abundance** | | ***P*-value** | ***Q*-value** |
|  | **40-50** | **20-30** |  |  |
| g_Prevotella | 0.0849 | 0.1581 | 0.004 | 0.674 |
| g_Agathobacter | 0.0131 | 0.0207 | 0.014 | 0.692 |
| f_Lachnospiraceae;g_uncultured | 0.0002 | 8.43E-05 | 0.022 | 0.692 |
| g_Bacteroides | 0.3252 | 0.2773 | 0.026 | 0.692 |
| g_Anaerostipes | 0.0131 | 0.0097 | 0.029 | 0.692 |
| g_Alloprevotella | 0.0016 | 0.0074 | 0.031 | 0.692 |
| g_Catenibacterium | 8.43E-05 | 0.0008 | 0.039 | 0.692 |
| **Feature** | **Average Relative Abundance** | | **P-value** | **Q-value** |
|  | **40-50** | **30-40** |  |  |
| g_[Ruminococcus]_torques_group | 0.0084 | 0.0055 | 0.007 | 0.708 |
| g_Butyricicoccus | 0.0056 | 0.0040 | 0.019 | 0.708 |
| g_Allisonella | 9.69E-05 | 0.0002 | 0.030 | 0.708 |
| g_Rikenellaceae_RC9_gut_group | 0.0000 | 3.28E-05 | 0.033 | 0.708 |
| g_Acidaminococcus | 1.19E-05 | 0.0004 | 0.041 | 0.708 |
| **Feature** | **Average Relative Abundance** | | **P-value** | **Q-value** |
|  | **40-50** | **<20** |  |  |
| g_Paraprevotella | 0.0062 | 2.61E-05 | <0.001 | <0.001 |
| g_[Eubacterium]_xylanophilum_group | 0.0004 | 7.26E-05 | <0.001 | 0.049 |
| g_Muribaculaceae | 0.0031 | 0.0000 | 0.002 | 0.087 |
| g_CAG-352 | 0.0090 | 0.0006 | 0.002 | 0.087 |
| g_Senegalimassilia | 0.0001 | 0.0000 | 0.003 | 0.090 |
| g_Holdemanella | 0.0028 | 0.0000 | 0.003 | 0.090 |
| g_Tyzzerella | 0.0032 | 0.0007 | 0.005 | 0.104 |
| g_Butyricicoccus | 0.0056 | 0.0028 | 0.005 | 0.104 |
| g_Erysipelotrichaceae_UCG-003 | 0.0052 | 0.0022 | 0.011 | 0.181 |
| g_Lachnospiraceae_UCG-001 | 0.0012 | 0.0003 | 0.013 | 0.181 |
| g_Fusobacterium | 0.0052 | 0.0001 | 0.014 | 0.181 |
| g_Coprococcus | 0.0071 | 0.0037 | 0.015 | 0.181 |
| g_[Eubacterium]_ruminantium_group | 0.0037 | 0.0010 | 0.015 | 0.181 |
| g_Lactococcus | 2.69E-05 | 0.0000 | 0.017 | 0.185 |
| g_Gastranaerophilales | 0.0002 | 0.0000 | 0.020 | 0.208 |
| g_Sellimonas | 7.61E-05 | 0.0007 | 0.026 | 0.248 |
| g_Megamonas | 0.0221 | 0.0070 | 0.034 | 0.307 |
| g_UCG-010 | 0.0007 | 0.0001 | 0.036 | 0.307 |
| g_Clostridia_vadinBB60_group | 0.0005 | 1.5E-05 | 0.042 | 0.340 |
| **Feature** | **Average Relative Abundance** | | ***P*-value** | ***Q*-value** |
|  | **>60** | **50-60** |  |  |
| g_Lachnospiraceae_UCG-010 | 0.0005 | 0.0015 | 0.002 | 0.154 |
| g_Negativibacillus | 9.89E-06 | 0.0005 | 0.002 | 0.154 |
| g_Sutterella | 0.0008 | 0.0042 | 0.003 | 0.154 |
| g_Lachnoclostridium | 0.0072 | 0.0170 | 0.010 | 0.393 |
| g_Allisonella | 0.0000 | 7.1E-05 | 0.020 | 0.625 |
| g_Family_XIII_UCG-001 | 1.47E-05 | 7.21E-05 | 0.028 | 0.692 |
| g_Fusobacterium | 0.0002 | 0.0029 | 0.037 | 0.692 |
| g_UCG-003 | 0.0009 | 0.0015 | 0.046 | 0.692 |
| **Feature** | **Average Relative Abundance** | | ***P*-value** | ***Q*-value** |
|  | **>60** | **20-30** |  |  |
| g_Sutterella | 0.0008 | 0.0080 | <0.001 | <0.001 |
| g_Negativibacillus | 9.89E-06 | 0.0004 | <0.001 | 0.005 |
| g_Senegalimassilia | 1.18E-05 | 0.0001 | 0.001 | 0.055 |
| g_Collinsella | 0.0026 | 0.0068 | 0.003 | 0.107 |
| g_Allisonella | 0.0000 | 0.0001 | 0.005 | 0.171 |
| g_Comamonas | 0.0000 | 3.06E-05 | 0.007 | 0.190 |
| g_Alloprevotella | 0.0012 | 0.0074 | 0.010 | 0.228 |
| g_Family_XIII_UCG-001 | 1.47E-05 | 6.07E-05 | 0.014 | 0.281 |
| g_Fusobacterium | 0.0002 | 0.0049 | 0.015 | 0.281 |
| g_Lachnospiraceae_UCG-010 | 0.0005 | 0.0011 | 0.022 | 0.367 |
| g_Akkermansia | 2.59E-05 | 0.0009 | 0.027 | 0.399 |
| g_Mitsuokella | 1.55E-05 | 0.0024 | 0.038 | 0.504 |
| g_UCG-003 | 0.0009 | 0.0013 | 0.040 | 0.504 |
| **Feature** | **Average Relative Abundance** | | ***P*-value** | ***Q*-value** |
|  | **>60** | **30-40** |  |  |
| g_Sutterella | 0.0008 | 0.0058 | <0.001 | <0.001 |
| g_Allisonella | 0.0000 | 0.0002 | <0.001 | <0.001 |
| g_Negativibacillus | 9.89E-06 | 0.0002 | <0.001 | 0.003 |
| g_Akkermansia | 2.59E-05 | 0.0007 | <0.001 | 0.035 |
| g_Fusobacterium | 0.0002 | 0.0110 | 0.003 | 0.081 |
| g_Lachnoclostridium | 0.0072 | 0.0142 | 0.009 | 0.183 |
| g_Family_XIII_UCG-001 | 1.47E-05 | 5.89E-05 | 0.009 | 0.183 |
| g_Collinsella | 0.0026 | 0.0089 | 0.009 | 0.183 |
| g_Enterorhabdus | 0.0000 | 4.68E-05 | 0.010 | 0.183 |
| g_Slackia | 0.0000 | 1.53E-05 | 0.0137 | 0.220 |
| g_Senegalimassilia | 1.18E-05 | 9.69E-05 | 0.022 | 0.318 |
| g_Mitsuokella | 1.55E-05 | 0.0010 | 0.024 | 0.318 |
| g_Prevotellaceae_NK3B31_group | 0.0002 | 0.0029 | 0.027 | 0.334 |
| g_Lachnospiraceae_UCG-010 | 0.0005 | 0.0010 | 0.032 | 0.334 |
| g_Rikenellaceae_RC9_gut_group | 0.0000 | 3.28E-05 | 0.033 | 0.334 |
| g_Coprobacter | 8.63E-05 | 0.0006 | 0.033 | 0.334 |
| f_Prevotellaceae;g_uncultured | 3.25E-05 | 0.0007 | 0.045 | 0.427 |
| **Feature** | **Average Relative Abundance** | | ***P*-value** | ***Q*-value** |
|  | **50-60** | **20-30** |  |  |
| g_Senegalimassilia | 1.65E-05 | 0.0001 | 0.002 | 0.181 |
| g_Lachnospiraceae_FCS020_group | 0.0002 | 0.0004 | 0.002 | 0.181 |
| g_Weissella | 7.39E-06 | 6.52E-05 | 0.011 | 0.580 |
| g_Howardella | 1.15E-05 | 5.1E-05 | 0.023 | 0.771 |
| g_Sutterella | 0.0042 | 0.0080 | 0.025 | 0.771 |
| **Feature** | **Average Relative Abundance** | | ***P*-value** | ***Q*-value** |
|  | **50-60** | **30-40** |  |  |
| g_Lachnospiraceae_FCS020_group | 0.0002 | 0.0004 | 0.006 | 0.449 |
| g_Haemophilus | 0.0004 | 0.0015 | 0.007 | 0.449 |
| g_Allisonella | 7.1E-05 | 0.0002 | 0.008 | 0.449 |
| g_Holdemania | 7.73E-05 | 2.91E-05 | 0.028 | 0.705 |
| g_Fusobacterium | 0.0029 | 0.0110 | 0.029 | 0.705 |
| g_Senegalimassilia | 1.65E-05 | 9.69E-05 | 0.034 | 0.705 |
| g_Prevotellaceae_NK3B31_group | 0.0003 | 0.0029 | 0.038 | 0.705 |
| **Feature** | **Average Relative Abundance** | | ***P*-value** | ***Q*-value** |
|  | **50-60** | **<20** |  |  |
| g_Paraprevotella | 0.0065 | 2.61E-05 | <0.001 | 0.062 |
| g_[Eubacterium]_xylanophilum_group | 0.0006 | 7.26E-05 | 0.002 | 0.128 |
| g_Muribaculaceae | 0.0014 | 0.0000 | 0.008 | 0.261 |
| g_Ruminococcus | 0.0124 | 0.0047 | 0.008 | 0.261 |
| g_Coprococcus | 0.0081 | 0.0037 | 0.011 | 0.261 |
| g_[Eubacterium]_ruminantium_group | 0.0048 | 0.0010 | 0.014 | 0.261 |
| g_Lachnospiraceae_UCG-001 | 0.0017 | 0.0003 | 0.017 | 0.261 |
| g_Clostridia_vadinBB60_group | 0.0003 | 1.5E-05 | 0.018 | 0.261 |
| g_Lachnoclostridium | 0.0170 | 0.0082 | 0.018 | 0.261 |
| g_Butyricicoccus | 0.0050 | 0.0028 | 0.019 | 0.261 |
| g_CAG-352 | 0.0053 | 0.0006 | 0.019 | 0.261 |
| g_Sellimonas | 5.18E-05 | 0.0007 | 0.021 | 0.261 |
| g_Lactococcus | 3.33E-05 | 0.0000 | 0.030 | 0.332 |
| g_Fusobacterium | 0.0029 | 0.0001 | 0.031 | 0.332 |
| g_Tyzzerella | 0.0029 | 0.0007 | 0.039 | 0.389 |
| g_Erysipelotrichaceae_UCG-003 | 0.0046 | 0.0022 | 0.041 | 0.389 |
| g_Eggerthella | 0.0002 | 0.0005 | 0.047 | 0.428 |
| **Feature** | **Average Relative Abundance** | | ***P*-value** | ***Q*-value** |
|  | **20-30** | **30-40** |  |  |
| g_Terrisporobacter | 4.34E-05 | 0.0001 | 0.006 | 0.668 |
| g_Holdemania | 6.41E-05 | 2.91E-05 | 0.012 | 0.668 |
| g_UCG-010 | 0.0002 | 0.0006 | 0.039 | 0.668 |
| g_Acidaminococcus | 9.75E-06 | 0.0004 | 0.040 | 0.668 |
| g_Dialister | 0.0128 | 0.0082 | 0.048 | 0.668 |
| **Feature** | **Average Relative Abundance** | | ***P*-value** | ***Q*-value** |
|  | **20-30** | **<20** |  |  |
| g_Paraprevotella | 0.0062 | 2.61E-05 | <0.001 | 0.001 |
| g_Muribaculaceae | 0.0016 | 0.0000 | <0.001 | 0.001 |
| g_Senegalimassilia | 0.0001 | 0.0000 | <0.001 | 0.010 |
| g_[Eubacterium]_xylanophilum_group | 0.0004 | 7.26E-05 | <0.001 | 0.023 |
| g_Clostridia_vadinBB60_group | 0.0002 | 1.5E-05 | 0.001 | 0.025 |
| g_Holdemanella | 0.0014 | 0.0000 | 0.001 | 0.025 |
| g_CAG-352 | 0.0060 | 0.0006 | 0.001 | 0.025 |
| g_Howardella | 5.1E-05 | 0.0000 | 0.001 | 0.025 |
| g_Coprococcus | 0.0082 | 0.0037 | 0.002 | 0.034 |
| g_Weissella | 6.52E-05 | 0.0000 | 0.003 | 0.051 |
| g_Comamonas | 3.06E-05 | 0.0000 | 0.007 | 0.096 |
| g_Ruminococcus | 0.0110 | 0.0047 | 0.008 | 0.096 |
| g_[Eubacterium]_ruminantium_group | 0.0040 | 0.0010 | 0.008 | 0.096 |
| g_Fournierella | 8.75E-05 | 0.0000 | 0.013 | 0.142 |
| g_Fusobacterium | 0.0049 | 0.0001 | 0.013 | 0.142 |
| g_Klebsiella | 0.0021 | 0.0005 | 0.017 | 0.165 |
| g_Catenibacterium | 0.0008 | 0.0000 | 0.018 | 0.165 |
| g_CAG-56 | 0.0020 | 0.0009 | 0.019 | 0.165 |
| g_Anaerostipes | 0.0097 | 0.0207 | 0.024 | 0.193 |
| g_Tyzzerella | 0.0018 | 0.0007 | 0.024 | 0.193 |
| g_Lachnospiraceae_UCG-001 | 0.0010 | 0.0003 | 0.030 | 0.228 |
| g_Prevotella | 0.1581 | 0.0631 | 0.034 | 0.249 |
| g_Gastranaerophilales | 0.0002 | 0.0000 | 0.038 | 0.252 |
| g_Alloprevotella | 0.0074 | 0.0016 | 0.038 | 0.252 |
| **Feature** | **Average Relative Abundance** | | ***P*-value** | ***Q*-value** |
|  | **30-40** | **<20** |  |  |
| g_Paraprevotella | 0.0056 | 2.61E-05 | <0.001 | <0.001 |
| g_[Eubacterium]_xylanophilum_group | 0.0004 | 7.26E-05 | <0.001 | 0.003 |
| g_Holdemanella | 0.0030 | 0.0000 | <0.001 | 0.024 |
| g_Howardella | 3.51E-05 | 0.0000 | <0.001 | 0.026 |
| g_Clostridia_vadinBB60_group | 0.0002 | 1.5E-05 | <0.001 | 0.031 |
| g_CAG-352 | 0.0049 | 0.0006 | 0.001 | 0.033 |
| g_Muribaculaceae | 0.0028 | 0.0000 | 0.002 | 0.038 |
| g_Lachnospiraceae_UCG-001 | 0.0014 | 0.0003 | 0.002 | 0.041 |
| g_Fusobacterium | 0.0110 | 0.0001 | 0.002 | 0.041 |
| g_Coprococcus | 0.0080 | 0.0037 | 0.003 | 0.049 |
| g_Fournierella | 5.81E-05 | 0.0000 | 0.004 | 0.058 |
| g_[Eubacterium]_ruminantium_group | 0.0042 | 0.0010 | 0.004 | 0.058 |
| g_Senegalimassilia | 9.69E-05 | 0.0000 | 0.006 | 0.071 |
| g_Ruminococcus | 0.0112 | 0.0047 | 0.006 | 0.071 |
| g_UCG-010 | 0.0006 | 0.0001 | 0.013 | 0.141 |
| g_Lachnoclostridium | 0.0142 | 0.0082 | 0.018 | 0.176 |
| g_Prevotellaceae_NK3B31_group | 0.0029 | 0.0000 | 0.018 | 0.176 |
| g_Coprobacter | 0.0006 | 6.13E-05 | 0.021 | 0.191 |
| g_Butyrivibrio | 0.0006 | 0.0000 | 0.023 | 0.201 |
| g_Sellimonas | 8.33E-05 | 0.0007 | 0.027 | 0.218 |
| g_Klebsiella | 0.0031 | 0.0005 | 0.029 | 0.226 |
| g_Rikenellaceae_RC9_gut_group | 3.28E-05 | 0.0000 | 0.033 | 0.244 |
| g_Acidaminococcus | 0.0004 | 0.0000 | 0.035 | 0.249 |
| g_uncultured | 0.0007 | 1.15E-05 | 0.038 | 0.259 |
| g_Anaerostipes | 0.0108 | 0.0207 | 0.040 | 0.260 |
| g_Weissella | 4.93E-05 | 0.0000 | 0.043 | 0.265 |
| g_Catenibacterium | 0.0003 | 0.0000 | 0.048 | 0.270 |
| g_Izemoplasmatales | 8.13E-06 | 0.0000 | 0.048 | 0.270 |
| g_Faecalibacterium | 0.0907 | 0.1295 | 0.048 | 0.270 |

**Supplementary Table 5. Associations of microbiota with age (continuous variable) at genus level adjusting for demographic factors including ethnicity, gender, ethnicity, BMI and habitation, living conditions including state of fatigue, degree of sleep deprivation, appearance of negative emotions, occurrence of oral ulcers and skin acne, and smoking frequency, and dietary habits including starch intake, protein intake and dietary preference using MaAsLin analysis**

| **Feature** | **Coefficient** | ***P*-value** | ***Q*-value** |
| --- | --- | --- | --- |
| g_Bifidobacterium | -0.053 | <0.001 | 0.005 |
| g_Erysipelatoclostridium | -0.022 | <0.001 | 0.009 |
| g_Sellimonas | -0.017 | 0.002 | 0.058 |
| g_Haemophilus | -0.034 | 0.005 | 0.130 |
| g_Butyricicoccus | 0.019 | 0.014 | 0.236 |
| g_Streptococcus | -0.023 | 0.017 | 0.236 |
| g_[Eubacterium]_xylanophilum_group | 0.020 | 0.016 | 0.236 |

MaAsLin, multivariate association with linear models.

**Supplementary Table 6. Associations of microbiota with BMI at genus level using t-test**

| **Feature** | **Average Relative Abundance** | | ***P*-value** | ***Q*-value** |
| --- | --- | --- | --- | --- |
|  | **Normal weight** | **Underweight** |  |  |
| g_Streptococcus | 0.0037 | 0.0013 | <0.001 | 0.081 |
| g_Holdemanella | 0.0028 | 0.0009 | 0.014 | 0.706 |
| g_[Ruminococcus]_gauvreauii_group | 0.0010 | 0.0006 | 0.023 | 0.706 |
| g_Lactobacillus | 0.0016 | 6.35E-05 | 0.029 | 0.706 |
| g_Enterobacter | 0.0009 | 8.92E-05 | 0.030 | 0.706 |
| g_Raoultella | 6.18E-05 | 0.0000 | 0.031 | 0.706 |
| g_Mitsuokella | 0.0009 | 1.27E-05 | 0.039 | 0.706 |
| g_Lachnoclostridium | 0.0133 | 0.0095 | 0.041 | 0.706 |
| g_Veillonella | 0.0017 | 0.0005 | 0.045 | 0.706 |
| **Feature** | **Average Relative Abundance** | | ***P*-value** | ***Q*-value** |
|  | **Normal weight** | **Overweight** |  |  |
| g_Fusicatenibacter | 0.0070 | 0.0051 | <0.001 | 0.102 |
| g_UCG-010 | 0.0006 | 0.0002 | 0.001 | 0.124 |
| g_Adlercreutzia | 0.0003 | 0.0002 | 0.006 | 0.248 |
| g_[Eubacterium]_xylanophilum_group | 0.0005 | 0.0002 | 0.007 | 0.248 |
| f_Ruminococcaceae;g_uncultured | 0.0010 | 0.0005 | 0.007 | 0.248 |
| g_Negativibacillus | 0.0004 | 0.0002 | 0.009 | 0.248 |
| g_Fenollaria | 1.5E-05 | 1.27E-06 | 0.013 | 0.321 |
| g_Holdemanella | 0.0028 | 0.0012 | 0.016 | 0.344 |
| g_Monoglobus | 0.0046 | 0.0032 | 0.020 | 0.371 |
| f_Oscillospiraceae;g_uncultured | 0.0005 | 0.0004 | 0.024 | 0.417 |
| g_Lachnospiraceae_UCG-010 | 0.0011 | 0.0008 | 0.034 | 0.535 |
| g_Oscillibacter | 0.0005 | 0.0003 | 0.039 | 0.545 |
| g_Coprobacter | 0.0004 | 0.0002 | 0.050 | 0.545 |
| **Feature** | **Average Relative Abundance** | | ***P*-value** | ***Q*-value** |
|  | **Underweight** | **Overweight** |  |  |
| g_Streptococcus | 0.0013 | 0.0032 | 0.006 | 0.502 |
| g_UCG-003 | 0.0017 | 0.0010 | 0.006 | 0.502 |
| g_Mitsuokella | 1.27E-05 | 0.0029 | 0.012 | 0.629 |
| g_Escherichia-Shigella | 0.0074 | 0.0167 | 0.028 | 0.629 |
| g_Holdemania | 0.0001 | 3.28E-05 | 0.029 | 0.629 |
| g_Fusicatenibacter | 0.0086 | 0.0051 | 0.031 | 0.629 |
| g_Allisonella | 5.68E-05 | 0.0003 | 0.033 | 0.629 |
| f_Oscillospiraceae;g_uncultured | 0.0006 | 0.0004 | 0.048 | 0.629 |

**Supplementary Table 7. Associations of microbiota with BMI (continuous variable) at genus level adjusting for demographic factors including ethnicity, age, gender, ethnicity and habitation, living conditions including state of fatigue, degree of sleep deprivation, appearance of negative emotions, occurrence of oral ulcers and skin acne, and smoking frequency, and dietary habits including starch intake, protein intake and dietary preference using MaAsLin analysis**

| **Feature** | **Coefficient** | ***P*-value** | ***Q*-value** |
| --- | --- | --- | --- |
| g_Flavonifractor | -0.154 | <0.001 | 0.003 |
| g_Eggerthella | -0.099 | <0.001 | 0.003 |
| g_Oscillibacter | -0.128 | <0.001 | 0.003 |
| g_Incertae_Sedis | -0.124 | <0.001 | 0.006 |
| g_Sellimonas | -0.070 | <0.001 | 0.014 |
| g_Erysipelatoclostridium | -0.079 | <0.001 | 0.014 |
| g_Holdemania | -0.067 | 0.002 | 0.017 |
| g_Allisonella | 0.085 | 0.001 | 0.017 |
| g_UBA1819 | -0.107 | 0.002 | 0.017 |
| g_Coprococcus | 0.111 | 0.004 | 0.042 |
| g_[Ruminococcus]_gnavus_group | -0.125 | 0.015 | 0.129 |
| g_Paraprevotella | 0.136 | 0.020 | 0.134 |
| g_Megasphaera | 0.101 | 0.021 | 0.134 |
| g_Dorea | 0.065 | 0.018 | 0.134 |
| g_Parasutterella | -0.131 | 0.017 | 0.134 |
| g_Prevotella | 0.162 | 0.022 | 0.136 |
| g_[Ruminococcus]_gauvreauii_group | 0.069 | 0.027 | 0.156 |
| g_Lachnospiraceae_UCG-003 | -0.071 | 0.034 | 0.186 |
| g_UCG-003 | -0.075 | 0.040 | 0.204 |
| g_Odoribacter | -0.089 | 0.046 | 0.222 |

MaAsLin, multivariate association with linear models.

**Supplementary Table 8. Associations of microbiota with ethnicity at genus level using t-test**

| **Feature** | **Average Relative Abundance** | | ***P*-value** | ***Q*-value** |
| --- | --- | --- | --- | --- |
|  | **Han** | **Others** |  |  |
| g_Parabacteroides | 0.0164 | 0.0087 | <0.001 | <0.001 |
| g_Bacteroides | 0.3141 | 0.2376 | <0.001 | 0.040 |
| g_Flavonifractor | 0.0009 | 0.0004 | 0.001 | 0.075 |
| g_Bilophila | 0.0010 | 0.0006 | 0.002 | 0.075 |
| g_Anaerotruncus | 4.69E-05 | 1.08E-05 | 0.004 | 0.113 |
| g_Dorea | 0.0060 | 0.0081 | 0.004 | 0.113 |
| g_Comamonas | 2.57E-05 | 2.26E-06 | 0.006 | 0.154 |
| g_Eisenbergiella | 7.3E-05 | 1.09E-05 | 0.007 | 0.154 |
| g_Oscillibacter | 0.0005 | 0.0003 | 0.009 | 0.157 |
| g_Faecalibacterium | 0.0943 | 0.1164 | 0.010 | 0.157 |
| g_[Ruminococcus]_gnavus_group | 0.0079 | 0.0037 | 0.010 | 0.157 |
| g_Catenibacterium | 0.0004 | 8.39E-05 | 0.016 | 0.221 |
| g_Tyzzerella | 0.0023 | 0.0013 | 0.018 | 0.236 |
| g_Escherichia-Shigella | 0.0133 | 0.0078 | 0.032 | 0.375 |
| g_Prevotella | 0.1090 | 0.1698 | 0.033 | 0.375 |
| g_Hungatella | 0.0004 | 6.44E-05 | 0.0378 | 0.396 |
| g_Fusicatenibacter | 0.0070 | 0.0056 | 0.040 | 0.396 |
| g_Howardella | 3.45E-05 | 1.38E-05 | 0.042 | 0.396 |
| f_Eggerthellaceae;g_uncultured | 1.74E-05 | 0.0000 | 0.044 | 0.400 |
| g_Coprococcus | 0.0075 | 0.0096 | 0.049 | 0.403 |
| g_UCG-010 | 0.0005 | 0.0003 | 0.050 | 0.403 |

**Supplementary Table 9. Associations of microbiota with ethnicity at genus level adjusting for demographic factors including ethnicity, age, gender, BMI and habitation, living conditions including state of fatigue, degree of sleep deprivation, appearance of negative emotions, occurrence of oral ulcers and skin acne, and smoking frequency, and dietary habits including starch intake, protein intake and dietary preference using MaAsLin analysis**

| **Feature** | **Group** | **Coefficient** | ***P*-value** | ***Q*-value** |
| --- | --- | --- | --- | --- |
| g_Parabacteroides | Others | -0.852 | 0.004 | 0.116 |
| g_Clostridia_UCG-014 | Others | 1.584 | 0.005 | 0.116 |
| g_Bacteroides | Others | -0.567 | 0.004 | 0.116 |
| g_Dorea | Others | 0.727 | 0.004 | 0.116 |
| g_Negativibacillus | Others | 0.675 | 0.015 | 0.249 |
| g_GCA-900066575 | Others | 0.522 | 0.015 | 0.249 |

MaAsLin, multivariate association with linear models.

**Supplementary Table 10. Statistical test results of inter-group and intra-group differences among age groups using Adonis and Anosim analysis and four distance matrices were applied: Unweighted Unifrac, Weighted Unifrac, Bray-curtis and Binary-jaccard**

| **Distance Matrices** | **Adonis** | | **Anosim** | |
| --- | --- | --- | --- | --- |
|  | **F. Model** | **Pr (> F)** | **Test Statistic** | ***p*-value** |
| **Unweighted Unifrac** | 2.5924 | 0.002 | -0.0287 | 0.907 |
| **Weighted Unifrac** | 3.6182 | 0.010 | 0.0170 | 0.241 |
| **Bray-curtis** | 2.4984 | 0.001 | 0.0273 | 0.134 |
| **Binary-jaccard** | 2.1724 | 0.001 | -0.0254 | 0.832 |

**Supplementary Table 11. Associations of microbiota with habitation at genus level using t-test**

| **Feature** | **Average Relative Abundance** | | ***P*-value** | ***Q*-value** |
| --- | --- | --- | --- | --- |
|  | **North** | **South** |  |  |
| g_Faecalibacterium | 0.0767 | 0.1045 | <0.001 | <0.001 |
| g_Fusobacterium | 0.0011 | 0.0081 | <0.001 | 0.014 |
| g_Erysipelotrichaceae_UCG-003 | 0.0027 | 0.0048 | 0.001 | 0.077 |
| g_Lachnospiraceae_ND3007_group | 0.0015 | 0.0022 | 0.003 | 0.143 |
| g_Fusicatenibacter | 0.0082 | 0.0063 | 0.010 | 0.302 |
| g_Dialister | 0.0148 | 0.0083 | 0.011 | 0.302 |
| g_Odoribacter | 0.0006 | 0.0010 | 0.013 | 0.307 |
| g_Raoultella | 1.8E-06 | 6.69E-05 | 0.024 | 0.398 |
| g_[Eubacterium]_ventriosum_group | 0.0020 | 0.0030 | 0.025 | 0.398 |
| f_Prevotellaceae;g_uncultured | 0.0001 | 0.0007 | 0.027 | 0.398 |
| g_Paraprevotella | 0.0040 | 0.0065 | 0.029 | 0.398 |
| g_Akkermansia | 0.0005 | 0.0028 | 0.029 | 0.398 |
| g_Erysipelatoclostridium | 6.9E-05 | 0.0005 | 0.030 | 0.398 |
| g_Hungatella | 6.78E-05 | 0.0004 | 0.035 | 0.410 |
| g_Butyrivibrio | 3.58E-05 | 0.0007 | 0.040 | 0.410 |
| g_Bifidobacterium | 0.0204 | 0.0118 | 0.042 | 0.410 |
| g_Clostridia_UCG-014 | 0.0035 | 0.0058 | 0.043 | 0.410 |

**Supplementary Table 12. Associations of microbiota with habitation at genus level adjusting for demographic factors including gender, ethnicity, Age and BMI, living conditions including state of fatigue, degree of sleep deprivation, appearance of negative emotions, occurrence of oral ulcers and skin acne, and smoking frequency, and dietary habits including starch intake, protein intake and dietary preference using MaAsLin analysis**

| **Feature** | **Group** | **Coefficient** | ***P*-value** | ***Q*-value** |
| --- | --- | --- | --- | --- |
| g_Klebsiella | South | -1.39 | <0.001 | <0.001 |
| g_Megasphaera | South | -1.205 | <0.001 | 0.003 |
| g_Collinsella | South | -1.376 | <0.001 | 0.003 |
| g_Bifidobacterium | South | -1.472 | <0.001 | 0.003 |
| g_Dialister | South | -1.270 | 0.006 | 0.114 |
| g_Clostridia_UCG-014 | South | 1.204 | 0.011 | 0.146 |
| g_Fusobacterium | South | 0.852 | 0.015 | 0.146 |
| g_Lachnospiraceae_UCG-003 | South | 0.579 | 0.013 | 0.146 |
| g_Erysipelotrichaceae_UCG-003 | South | 0.785 | 0.014 | 0.146 |
| g_Enterobacter | South | -0.510 | 0.014 | 0.146 |
| g_Paraprevotella | South | 0.989 | 0.017 | 0.151 |
| g_Adlercreutzia | South | 0.451 | 0.029 | 0.235 |
| g_Clostridia_vadinBB60_group | South | 0.445 | 0.037 | 0.249 |
| g_Sutterella | South | -0.911 | 0.035 | 0.249 |
| g_Akkermansia | South | 0.560 | 0.038 | 0.249 |

MaAsLin, multivariate association with linear models.

**Supplementary Table 13. Alpha diversity between sleep deprivation groups and the statistics results using Kruskal-Wallis analysis**

| **Group** | | **Chao 1 index** | | | | **Shannon index** | | |
| --- | --- | --- | --- | --- | --- | --- | --- | --- |
| **Group 1** | **Group 2** | **H** | **P-value** | **Q-value** | **H** | | **P-value** | **Q-value** |
| **Less** | **More** | 0.5255 | 0.469 | 0.588 | 0.0743 | | 0.785 | 0.785 |
| **Less** | **Normal** | 0.2929 | 0.589 | 0.588 | 0.5782 | | 0.447 | 0.671 |
| **More** | **Normal** | 1.1224 | 0.289 | 0.588 | 0.8142 | | 0.367 | 0.671 |

**Supplementary Table 14. Associations of microbiota with degree of sleep deprivation at genus level using t-test**

| **Feature** | **Average Relative Abundance** | | ***P*-value** | ***Q*-value** |
| --- | --- | --- | --- | --- |
|  | **More** | **Normal** |  |  |
| g_Mitsuokella | 0.0033 | 0.0002 | 0.021 | 0.703 |
| g_UCG-005 | 0.0030 | 0.0055 | 0.033 | 0.703 |
| g_Subdoligranulum | 0.0181 | 0.0143 | 0.034 | 0.703 |
| g_Allisonella | 0.0002 | 6.669E-05 | 0.038 | 0.703 |
| g_Collinsella | 0.0107 | 0.0054 | 0.040 | 0.703 |
| **Feature** | **Average Relative Abundance** | | ***P*-value** | ***Q*-value** |
|  | **More** | **Less** |  |  |
| g_Parabacteroides | 0.0129 | 0.0179 | 0.018 | 0.765 |
| g_[Eubacterium]_hallii_group | 0.0124 | 0.0084 | 0.019 | 0.765 |
| g_Parasutterella | 0.0050 | 0.0078 | 0.041 | 0.765 |
| g_Collinsella | 0.0107 | 0.0056 | 0.044 | 0.765 |
| g_Butyricimonas | 0.0004 | 0.0007 | 0.048 | 0.765 |
| **Feature** | **Average Relative Abundance** | | ***P*-value** | ***Q*-value** |
|  | **Normal** | **Less** |  |  |
| g_Bacteroides | 0.2809 | 0.3247 | 0.016 | 0.713 |
| g_Allisonella | 6.669E-05 | 0.0001 | 0.022 | 0.713 |
| g_Lachnospiraceae_UCG-003 | 0.0015 | 0.0007 | 0.032 | 0.713 |
| g_Comamonas | 2.336E-06 | 3.172E-05 | 0.033 | 0.713 |

**Supplementary Table 15. Associations of microbiota with degree of sleep deprivation at genus level adjusting for demographic factors including gender, ethnicity, Age, BMI and habitation, living conditions including state of fatigue, appearance of negative emotions, occurrence of oral ulcers and skin acne, and smoking frequency, and dietary habits including starch intake, protein intake and dietary preference using MaAsLin analysis**

| **Feature** | **Group** | **Coefficient** | ***P*-value** | ***Q*-value** |
| --- | --- | --- | --- | --- |
| g_ [Eubacterium]_hallii_group | More | 0.783 | <0.001 | 0.125 |
| g_ Bifidobacterium | More | 1.367 | 0.001 | 0.125 |
| g_ Parabacteroides | Less | 0.738 | 0.002 | 0.153 |

MaAsLin, multivariate association with linear models. Normal (average amount of sleep achieved) was set to be reference.

**Supplementary Table 16. Associations of microbiota with state of fatigue at genus level using t-test**

| **Feature** | **Average Relative Abundance** | | ***P*-value** | ***Q*-value** |
| --- | --- | --- | --- | --- |
|  | **Sometimes** | **Normal** |  |  |
| g_Raoultella | 5.6E-05 | 0.0000 | 0.013 | 0.699 |
| f_Prevotellaceae;g_uncultured | 0.0009 | 7.76E-05 | 0.014 | 0.699 |
| g_UCG-005 | 0.0029 | 0.0060 | 0.016 | 0.699 |
| g_[Ruminococcus]_torques_group | 0.0072 | 0.0055 | 0.023 | 0.699 |
| g_Lactococcus | 7.68E-05 | 2.05E-05 | 0.025 | 0.699 |
| g_Negativibacillus | 0.0004 | 0.0002 | 0.037 | 0.699 |
| g_Marvinbryantia | 4.48E-05 | 9.93E-05 | 0.039 | 0.699 |
| g_Fusicatenibacter | 0.0061 | 0.0076 | 0.045 | 0.699 |
| g_Lachnospiraceae_FCS020_group | 0.0003 | 0.0005 | 0.047 | 0.699 |
| g_Faecalitalea | 7.42E-05 | 1.17E-05 | 0.049 | 0.699 |
| **Feature** | **Average Relative Abundance** | | ***P*-value** | ***Q*-value** |
|  | **Sometimes** | **Always** |  |  |
| g_Fusicatenibacter | 0.0061 | 0.0083 | 0.006 | 0.766 |
| g_Lachnospiraceae_UCG-004 | 0.0042 | 0.0055 | 0.026 | 0.766 |
| g_Sellimonas | 0.0001 | 3.24E-05 | 0.030 | 0.766 |
| g_Catenibacterium | 0.0005 | 9.19E-05 | 0.042 | 0.766 |
| g_Lachnospira | 0.0136 | 0.0180 | 0.046 | 0.766 |
| **Feature** | **Average Relative Abundance** | | ***P*-value** | ***Q*-value** |
|  | **Normal** | **Always** |  |  |
| g_Sellimonas | 0.0002 | 3.24E-05 | 0.026 | 0.731 |
| g_Family_XIII_AD3011_group | 2.39E-05 | 4.09E-06 | 0.043 | 0.731 |

**Supplementary Table 17. Associations of microbiota with appearance of negative emotions at genus level using t-test**

| **Feature** | **Average Relative Abundance** | | ***P*-value** | ***Q*-value** |
| --- | --- | --- | --- | --- |
|  | **Negative emotions** | **Normal** |  |  |
| g_Negativibacillus | 0.0005 | 0.0002 | 0.001 | 0.153 |
| g_Tyzzerella | 0.0015 | 0.0026 | 0.001 | 0.784 |
| g_Eggerthella | 0.0001 | 0.0003 | 0.014 | 0.784 |
| g_Family_XIII_AD3011_group | 8.57E-06 | 4.24E-05 | 0.049 | 0.784 |
| g_Subdoligranulum | 0.0173 | 0.0147 | 0.050 | 0.784 |

**Supplementary Table 18. Associations of microbiota with appearance of negative emotions at genus level adjusting for demographic factors including gender, ethnicity, Age, BMI and habitation, living conditions including state of fatigue, degree of sleep deprivation, occurrence of oral ulcers and skin acne, and smoking frequency, and dietary habits including starch intake, protein intake and dietary preference using MaAsLin analysis**

| **Feature** | **Group** | **Coefficient** | ***P*-value** | ***Q*-value** |
| --- | --- | --- | --- | --- |
| g_ Negativibacillus | Normal | -0.687 | <0.001 | 0.037 |

MaAsLin, multivariate association with linear models.

**Supplementary Table 19. Associations of microbiota with occurrence of oral ulcers and skin acne at genus level using t-test**

| **Feature** | **Average Relative Abundance** | | ***P*-value** | ***Q*-value** |
| --- | --- | --- | --- | --- |
|  | **Seldom** | **Sometimes** |  |  |
| g_Gastranaerophilales | 0.0002 | 5.07E-05 | 0.022 | 0.822 |
| g_Blautia | 0.0294 | 0.0254 | 0.028 | 0.822 |
| g_[Eubacterium]_hallii_group | 0.0112 | 0.0086 | 0.028 | 0.822 |
| g_Paraprevotella | 0.0045 | 0.0076 | 0.036 | 0.822 |
| g_Parasutterella | 0.0080 | 0.0054 | 0.037 | 0.822 |
| g_Comamonas | 6.43E-06 | 4.48E-05 | 0.044 | 0.822 |
| **Feature** | **Average Relative Abundance** | | ***P*-value** | ***Q*-value** |
|  | **Seldom** | **Always** |  |  |
| g_Marvinbryantia | 6.97E-05 | 1.27E-05 | 0.002 | 0.199 |
| g_Dialister | 0.0108 | 0.0052 | 0.002 | 0.199 |
| g_Catenibacterium | 0.0003 | 1.5E-06 | 0.004 | 0.231 |
| g_[Eubacterium]_ventriosum_group | 0.0029 | 0.0017 | 0.009 | 0.337 |
| g_Fusobacterium | 0.0063 | 0.0013 | 0.013 | 0.337 |
| g_Enterorhabdus | 3.83E-05 | 0.0000 | 0.015 | 0.337 |
| g_Turicibacter | 0.0005 | 0.0001 | 0.016 | 0.337 |
| g_Escherichia-Shigella | 0.0153 | 0.0080 | 0.016 | 0.337 |
| g_Veillonella | 0.0016 | 0.0004 | 0.019 | 0.368 |
| g_Fusicatenibacter | 0.0064 | 0.0089 | 0.030 | 0.518 |
| g_Incertae_Sedis | 0.0011 | 0.0007 | 0.036 | 0.559 |
| g_Erysipelotrichaceae_UCG-003 | 0.0050 | 0.0031 | 0.043 | 0.600 |
| o_Bacteroidales;f_uncultured;g_uncultured | 6.14E-05 | 6.68E-06 | 0.048 | 0.600 |
| **Feature** | **Average Relative Abundance** | | ***P*-value** | ***Q*-value** |
|  | **Sometimes** | **Always** |  |  |
| g_Marvinbryantia | 7.11E-05 | 1.27E-05 | 0.003 | 0.415 |
| g_Fusobacterium | 0.0082 | 0.0013 | 0.007 | 0.527 |
| g_Enterorhabdus | 5.12E-05 | 0.0000 | 0.016 | 0.527 |
| g_Bacteroides | 0.2868 | 0.3410 | 0.018 | 0.527 |
| g_Family_XIII_AD3011_group | 2.34E-05 | 2.17E-06 | 0.019 | 0.527 |
| g_Gordonibacter | 1.53E-05 | 2.34E-06 | 0.020 | 0.527 |
| g_Dialister | 0.0105 | 0.0052 | 0.023 | 0.527 |
| g_[Eubacterium]_ventriosum_group | 0.0029 | 0.0017 | 0.028 | 0.527 |
| g_Catenibacterium | 0.0006 | 1.5E-06 | 0.031 | 0.527 |
| g_Lachnoclostridium | 0.0145 | 0.0101 | 0.032 | 0.527 |
| g_Fusicatenibacter | 0.0065 | 0.0089 | 0.046 | 0.555 |
| g_UCG-010 | 0.0006 | 0.0002 | 0.046 | 0.555 |
| g_Prevotella | 0.1396 | 0.0875 | 0.048 | 0.555 |

**Supplementary Table 20. Statistical test results of inter-group and intra-group differences among smoking frequency groups using Adonis and Anosim analysis and four distance matrices were applied: Unweighted Unifrac, Weighted Unifrac, Bray-curtis and Binary-jaccard**

| **Distance Matrices** | **Adonis** | | **Anosim** | |
| --- | --- | --- | --- | --- |
|  | **F. Model** | **Pr (> F)** | **R-value** | ***p*-value** |
| **Unweighted Unifrac** | 1.8392 | 0.010 | 0.0489 | 0.07 |
| **Weighted Unifrac** | 0.9657 | 0.441 | 0.0668 | 0.021 |
| **Bray-curtis** | 1.2856 | 0.029 | 0.0847 | 0.012 |
| **Binary-jaccard** | 1.3064 | 0.002 | 0.0712 | 0.045 |

**Supplementary Table 21. Alpha diversity between smoking frequency groups and the statistics results using Kruskal-Wallis analysis**

| **Group** | | **Chao 1 index** | | | | **Shannon index** | | |
| --- | --- | --- | --- | --- | --- | --- | --- | --- |
| **Group 1** | **Group 2** | **H** | **P-value** | **Q-value** | **H** | | ***P*-value** | ***Q*-value** |
| **Always** | **Never** | 7.5386 | 0.006 | 0.018 | 4.3423 | | 0.037 | 0.112 |
| **Always** | **Sometimes** | 0.5407 | 0.462 | 0.462 | 0.0338 | | 0.854 | 0.854 |
| **Never** | **Sometimes** | 2.9401 | 0.086 | 0.130 | 3.0484 | | 0.081 | 0.121 |

**Supplementary Table 22. Associations of microbiota with smoking frequency at genus level using t-test**

| **Feature** | **Average Relative Abundance** | | ***P*-value** | ***Q*-value** |
| --- | --- | --- | --- | --- |
|  | **Never** | **Sometimes** |  |  |
| g_[Eubacterium]_siraeum_group | 0.0023 | 0.0003 | <0.001 | <0.001 |
| g_Monoglobus | 0.0045 | 0.0020 | <0.001 | <0.001 |
| g_[Eubacterium]_ruminantium_group | 0.0048 | 0.0015 | <0.001 | 0.001 |
| g_UCG-010 | 0.0006 | 0.0001 | <0.001 | 0.002 |
| g_Lachnospiraceae_ND3007_group | 0.0022 | 0.0011 | <0.001 | 0.002 |
| g_Gastranaerophilales | 0.0002 | 0.0000 | <0.001 | 0.008 |
| g_Lachnospiraceae_NK4A136_group | 0.0113 | 0.0060 | <0.001 | 0.017 |
| g_Catenibacterium | 0.0005 | 0.0000 | <0.001 | 0.017 |
| g_Megasphaera | 0.0018 | 0.0003 | 0.003 | 0.053 |
| g_Fenollaria | 1.48E-05 | 0.0000 | 0.004 | 0.062 |
| g_Prevotellaceae_NK3B31_group | 0.0016 | 0.0001 | 0.006 | 0.086 |
| g_CAG-352 | 0.0062 | 0.0020 | 0.006 | 0.090 |
| g_Eggerthella | 0.0002 | 7.99E-05 | 0.008 | 0.103 |
| g_RF39 | 2.91E-05 | 0.0000 | 0.009 | 0.103 |
| g_GCA-900066575 | 0.0003 | 0.0001 | 0.009 | 0.103 |
| g_Coprobacter | 0.0004 | 0.0001 | 0.013 | 0.134 |
| g_Lachnospiraceae_UCG-010 | 0.0011 | 0.0007 | 0.017 | 0.166 |
| o_Bacteroidales;f_uncultured;g_uncultured | 0.0001 | 0.0000 | 0.019 | 0.173 |
| g_[Eubacterium]_eligens_group | 0.0081 | 0.0047 | 0.019 | 0.173 |
| g_Lactobacillus | 0.0010 | 0.0002 | 0.027 | 0.225 |
| g_Enterobacter | 0.0009 | 0.0001 | 0.028 | 0.225 |
| g_Family_XIII_UCG-001 | 6.31E-05 | 2.61E-05 | 0.031 | 0.243 |
| g_Rikenellaceae_RC9_gut_group | 2.88E-05 | 0.0000 | 0.034 | 0.252 |
| g_Clostridia_UCG-014 | 0.0055 | 0.0029 | 0.038 | 0.270 |
| g_Butyricicoccus | 0.0046 | 0.0036 | 0.044 | 0.288 |
| g_UCG-003 | 0.0013 | 0.0010 | 0.044 | 0.288 |
| **Feature** | **Average Relative Abundance** | | ***P*-value** | ***Q*-value** |
|  | **Never** | **Always** |  |  |
| g_Lachnospiraceae_NK4A136_group | 0.0113 | 0.0033 | <0.001 | <0.001 |
| g_UCG-010 | 0.0006 | 5.72E-05 | <0.001 | <0.001 |
| g_[Eubacterium]_siraeum_group | 0.0023 | 0.0004 | <0.001 | <0.001 |
| f_Ruminococcaceae;g_uncultured | 0.0010 | 0.0003 | <0.001 | <0.001 |
| g_Marvinbryantia | 7.37E-05 | 8.19E-06 | <0.001 | <0.001 |
| g_Christensenellaceae_R-7_group | 0.0054 | 0.0017 | <0.001 | 0.002 |
| g_GCA-900066575 | 0.0003 | 0.0001 | <0.001 | 0.005 |
| g_Haemophilus | 0.0015 | 0.0004 | <0.001 | 0.007 |
| g_[Eubacterium]_xylanophilum_group | 0.0005 | 0.0002 | <0.001 | 0.008 |
| g_Catenibacterium | 0.0005 | 0.0000 | <0.001 | 0.013 |
| g_Muribaculaceae | 0.0026 | 0.0007 | 0.001 | 0.018 |
| g_Enterorhabdus | 4.11E-05 | 0.0000 | 0.001 | 0.018 |
| g_[Eubacterium]_eligens_group | 0.0081 | 0.0039 | 0.001 | 0.018 |
| g_UCG-005 | 0.0039 | 0.0018 | 0.003 | 0.033 |
| g_[Eubacterium]_ruminantium_group | 0.0048 | 0.0019 | 0.003 | 0.033 |
| g_Lachnospiraceae_FCS020_group | 0.0004 | 0.0002 | 0.003 | 0.033 |
| g_Lachnospiraceae_ND3007_group | 0.0022 | 0.0013 | 0.004 | 0.038 |
| g_UCG-003 | 0.0013 | 0.0008 | 0.004 | 0.038 |
| g_Eisenbergiella | 6.8E-05 | 8.61E-06 | 0.007 | 0.065 |
| g_Butyrivibrio | 0.0004 | 4.07E-05 | 0.008 | 0.071 |
| g_Akkermansia | 0.0019 | 0.0002 | 0.009 | 0.071 |
| g_Faecalibacterium | 0.1007 | 0.0778 | 0.011 | 0.083 |
| f_Oscillospiraceae;g_uncultured | 0.0005 | 0.0003 | 0.013 | 0.090 |
| g_Fenollaria | 1.48E-05 | 1.85E-06 | 0.014 | 0.090 |
| g_Senegalimassilia | 0.0001 | 3.38E-05 | 0.014 | 0.090 |
| g_Clostridia_vadinBB60_group | 0.0003 | 8.73E-05 | 0.014 | 0.090 |
| g_Frisingicoccus | 2.56E-05 | 0.0000 | 0.014 | 0.090 |
| g_Phocea | 1.88E-05 | 4.67E-06 | 0.017 | 0.106 |
| g_Subdoligranulum | 0.0162 | 0.0118 | 0.020 | 0.115 |
| g_UCG-002 | 0.0069 | 0.0043 | 0.023 | 0.132 |
| g_Lachnospiraceae_UCG-004 | 0.0047 | 0.0033 | 0.025 | 0.139 |
| g_Lachnospiraceae_UCG-001 | 0.0013 | 0.0008 | 0.027 | 0.143 |
| g_Lachnospiraceae_UCG-010 | 0.0011 | 0.0008 | 0.036 | 0.186 |
| g_Coprobacillus | 7.07E-06 | 0.0000 | 0.040 | 0.194 |
| g_Romboutsia | 0.0026 | 0.0058 | 0.041 | 0.194 |
| g_Coprobacter | 0.0004 | 0.0001 | 0.041 | 0.194 |
| g_Incertae_Sedis | 0.0013 | 0.0008 | 0.046 | 0.213 |
| g_Gastranaerophilales | 0.0002 | 6.59E-05 | 0.048 | 0.217 |

**Supplementary Table 23. Statistical test results of inter-group and intra-group differences among starch intake groups using Adonis and Anosim analysis and four distance matrices were applied: Unweighted Unifrac, Weighted Unifrac, Bray-curtis and Binary-jaccard**

| **Distance Matrices** | **Adonis** | | **Anosim** | |
| --- | --- | --- | --- | --- |
|  | **F. Model** | **Pr (> F)** | **R-value** | ***p*-value** |
| **Unweighted Unifrac** | 1.2536 | 0.097 | -0.0584 | 0.962 |
| **Weighted Unifrac** | 1.5615 | 0.098 | 0.0102 | 0.384 |
| **Bray-curtis** | 1.2430 | 0.008 | -0.0321 | 0.822 |
| **Binary-jaccard** | 1.2111 | 0.012 | -0.0962 | 0.997 |

**Supplementary Table 24. Alpha diversity between starch intake groups and the statistics results using Kruskal-Wallis analysis**

| **Group** | | **Chao 1 index** | | | **Shannon index** | | | |  |
| --- | --- | --- | --- | --- | --- | --- | --- | --- | --- |
| **Group 1** | **Group 2** | **H** | ***P*-value** | ***Q*-value** | | **H** | ***P*-value** | ***Q*-value** | |
| Cereal | Flour-based foods | 2.0543 | 0.152 | 0.422 | | 10.3924 | 0.001 | 0.008 | |
| Cereal | High-starch foods | 0.2595 | 0.611 | 0.733 | | 0.5207 | 0.471 | 0.471 | |
| Cereal | Rice | 3.7038 | 0.054 | 0.326 | | 6.8759 | 0.009 | 0.026 | |
| Flour-based foods | High-starch foods | 1.1601 | 0.281 | 0.422 | | 2.1760 | 0.140 | 0.210 | |
| Flour-based foods | Rice | 0.0629 | 0.802 | 0.802 | | 2.8096 | 0.093 | 0.187 | |
| High-starch foods | Rice | 1.4583 | 0.227 | 0.422 | | 1.7021 | 0.192 | 0.230 | |

**Supplementary Table 25. Associations of microbiota with starch intake at genus level using t-test**

| **Feature** | **Average Relative Abundance** | | ***P*-value** | ***Q*-value** |
| --- | --- | --- | --- | --- |
|  | **Flour-based** | **Cereal** |  |  |
| g_Subdoligranulum | 0.0141 | 0.0233 | 0.002 | 0.369 |
| g_Streptococcus | 0.0040 | 0.0011 | 0.011 | 0.598 |
| g_Escherichia-Shigella | 0.0156 | 0.0029 | 0.014 | 0.598 |
| g_[Eubacterium]_eligens_group | 0.0049 | 0.0110 | 0.020 | 0.598 |
| g_[Ruminococcus]_gnavus_group | 0.0052 | 0.0011 | 0.022 | 0.598 |
| g_Coprococcus | 0.0069 | 0.0121 | 0.029 | 0.598 |
| g_Faecalibacterium | 0.0782 | 0.1050 | 0.032 | 0.598 |
| g_Lachnospiraceae_ND3007_group | 0.0014 | 0.0033 | 0.033 | 0.598 |
| g_Veillonella | 0.0018 | 0.0003 | 0.034 | 0.598 |
| o_Rhodospirillales;f_uncultured;g_uncultured | 0.0006 | 0.0044 | 0.047 | 0.623 |
| **Feature** | **Average Relative Abundance** | | ***P*-value** | ***Q*-value** |
|  | **Flour-based** | **Rice** |  |  |
| g_Adlercreutzia | 9.16E-05 | 0.0010 | <0.001 | 0.002 |
| g_Lachnospiraceae_UCG-003 | 0.0003 | 0.0032 | <0.001 | 0.011 |
| g_Howardella | 3.99E-06 | 0.0001 | <0.001 | 0.016 |
| g_Allisonella | 3.86E-05 | 0.0006 | <0.001 | 0.033 |
| g_Marvinbryantia | 1.55E-05 | 0.0002 | 0.001 | 0.046 |
| g_Eggerthella | 8.42E-05 | 0.0009 | 0.002 | 0.050 |
| g_Gastranaerophilales | 6.83E-06 | 0.0009 | 0.003 | 0.069 |
| f_Prevotellaceae;g_uncultured | 3.83E-05 | 0.0046 | 0.005 | 0.102 |
| g_Faecalibacterium | 0.0782 | 0.0651 | 0.005 | 0.102 |
| g_Parabacteroides | 0.0111 | 0.0219 | 0.009 | 0.147 |
| o_Rhodospirillales;f_uncultured;g_uncultured | 0.0006 | 0.0088 | 0.009 | 0.147 |
| g_Frisingicoccus | 0.0000 | 0.0002 | 0.016 | 0.230 |
| o_Bacteroidales;f_uncultured;g_uncultured | 2.62E-06 | 0.0011 | 0.018 | 0.235 |
| g_Raoultella | 0.0000 | 0.0005 | 0.019 | 0.235 |
| g_Clostridium_sensu_stricto_1 | 0.0018 | 0.0113 | 0.023 | 0.241 |
| g_[Eubacterium]_eligens_group | 0.0049 | 0.0122 | 0.024 | 0.241 |
| g_Bifidobacterium | 0.0344 | 0.0243 | 0.024 | 0.241 |
| g_Enterorhabdus | 6.80E-05 | 0.0002 | 0.027 | 0.254 |
| g_Odoribacter | 0.0006 | 0.0028 | 0.029 | 0.263 |
| g_Lachnospiraceae_ND3007_group | 0.0014 | 0.0031 | 0.042 | 0.345 |
| g_Butyrivibrio | 2.35E-05 | 0.0060 | 0.043 | 0.345 |
| g_Blautia | 0.0243 | 0.0200 | 0.044 | 0.345 |
| **Feature** | **Average Relative Abundance** | | ***P*-value** | ***Q*-value** |
|  | **Flour-based** | **High-starch** |  |  |
| g_Megamonas | 0.0130 | 0.0002 | <0.001 | 0.029 |
| g_Bifidobacterium | 0.0344 | 0.0059 | 0.009 | 0.355 |
| g_Bilophila | 0.0010 | 0.0003 | 0.010 | 0.355 |
| g_Megasphaera | 0.0018 | 0.0000 | 0.013 | 0.355 |
| g_Veillonella | 0.0018 | 8.22E-05 | 0.013 | 0.355 |
| g_Haemophilus | 0.0018 | 0.0002 | 0.013 | 0.355 |
| g_Senegalimassilia | 9.13E-05 | 0.0000 | 0.018 | 0.388 |
| g_[Ruminococcus]_gnavus_group | 0.0052 | 0.0009 | 0.021 | 0.391 |
| g_Family_XIII_AD3011_group | 1.86E-05 | 0.0000 | 0.023 | 0.391 |
| g_Alistipes | 0.0044 | 0.0016 | 0.029 | 0.441 |
| f_Ruminococcaceae;g_uncultured | 0.0009 | 0.0003 | 0.037 | 0.484 |
| g_Agathobacter | 0.0238 | 0.0097 | 0.038 | 0.484 |
| g_[Eubacterium]_ventriosum_group | 0.0023 | 0.0009 | 0.045 | 0.493 |
| g_Enterococcus | 0.0001 | 0.0000 | 0.045 | 0.493 |
| **Feature** | **Average Relative Abundance** | | ***P*-value** | ***Q*-value** |
|  | **Cereal** | **Rice** |  |  |
| g_[Ruminococcus]_gnavus_group | 0.0012 | 0.0082 | <0.001 | <0.001 |
| g_Escherichia-Shigella | 0.0029 | 0.0129 | <0.001 | <0.001 |
| g_Fusobacterium | 0.0004 | 0.0072 | <0.001 | 0.002 |
| g_Streptococcus | 0.0011 | 0.0035 | <0.001 | 0.004 |
| g_[Eubacterium]_ventriosum_group | 0.0016 | 0.0029 | 0.002 | 0.070 |
| g_Comamonas | 0.0000 | 2.67E-05 | 0.003 | 0.075 |
| g_Subdoligranulum | 0.0233 | 0.0154 | 0.004 | 0.099 |
| g_Faecalitalea | 2.49E-06 | 6.34E-05 | 0.007 | 0.126 |
| g_Veillonella | 0.0003 | 0.0015 | 0.007 | 0.126 |
| f_Prevotellaceae;g_uncultured | 6.34E-05 | 0.0007 | 0.008 | 0.126 |
| g_Clostridium_sensu_stricto_1 | 0.0013 | 0.0035 | 0.008 | 0.126 |
| g_[Ruminococcus]_torques_group | 0.0045 | 0.0070 | 0.010 | 0.136 |
| g_Hungatella | 1.18E-05 | 0.0004 | 0.011 | 0.148 |
| g_Erysipelatoclostridium | 6.42E-06 | 0.0004 | 0.012 | 0.148 |
| o_Bacteroidales;f_uncultured;g_uncultured | 0.0000 | 0.0001 | 0.016 | 0.184 |
| g_Raoultella | 0.0000 | 6.18E-05 | 0.019 | 0.205 |
| g_Turicibacter | 0.0001 | 0.0004 | 0.028 | 0.280 |
| g_Sutterella | 0.0038 | 0.0061 | 0.030 | 0.288 |
| g_Fusicatenibacter | 0.0087 | 0.0064 | 0.034 | 0.304 |
| g_Rikenellaceae_RC9_gut_group | 0.0000 | 2.79E-05 | 0.039 | 0.330 |
| g_Coprococcus | 0.0121 | 0.0075 | 0.043 | 0.343 |
| g_Parabacteroides | 0.0118 | 0.0164 | 0.045 | 0.343 |
| g_Erysipelotrichaceae_UCG-003 | 0.0030 | 0.0045 | 0.046 | 0.343 |
| **Feature** | **Average Relative Abundance** | | ***P*-value** | ***Q*-value** |
|  | **Cereal** | **High-starch** |  |  |
| g_Megamonas | 0.0243 | 0.0002 | 0.001 | 0.168 |
| g_Alistipes | 0.0068 | 0.0016 | 0.004 | 0.291 |
| g_Bilophila | 0.0008 | 0.0003 | 0.026 | 0.690 |
| g_[Eubacterium]_coprostanoligenes_group | 0.0069 | 0.0033 | 0.038 | 0.690 |
| g_Agathobacter | 0.0242 | 0.0097 | 0.040 | 0.690 |
| g__Oscillibacter | 0.0008 | 0.0003 | 0.044 | 0.690 |
| **Feature** | **Average Relative Abundance** | | ***P*-value** | ***Q*-value** |
|  | **Rice** | **High-starch** |  |  |
| g_Megamonas | 0.0188 | 0.0002 | <0.001 | <0.001 |
| g_Holdemanella | 0.0026 | 6.79E-05 | <0.001 | <0.001 |
| g_[Ruminococcus]_gnavus_group | 0.0082 | 0.0009 | <0.001 | <0.001 |
| g_Senegalimassilia | 0.0001 | 0.0000 | <0.001 | <0.001 |
| g_Fusobacterium | 0.0072 | 0.0000 | <0.001 | <0.001 |
| g_Alloprevotella | 0.0049 | 0.0000 | <0.001 | <0.001 |
| g_Alistipes | 0.0065 | 0.0016 | <0.001 | <0.001 |
| g_Haemophilus | 0.0013 | 0.0002 | <0.001 | 0.002 |
| g_Phocea | 1.47E-05 | 0.0000 | <0.001 | 0.002 |
| g_[Eubacterium]_ventriosum_group | 0.0029 | 0.0009 | <0.001 | 0.003 |
| g_Megasphaera | 0.0019 | 0.0000 | <0.001 | 0.004 |
| g_Catenibacterium | 0.0003 | 0.0000 | <0.001 | 0.010 |
| f_Ruminococcaceae;g_uncultured | 0.0009 | 0.0003 | <0.001 | 0.010 |
| g_Bilophila | 0.0009 | 0.0003 | <0.001 | 0.011 |
| g_Veillonella | 0.0015 | 8.22E-05 | 0.001 | 0.012 |
| g_Enterorhabdus | 3.63E-05 | 0.0000 | 0.002 | 0.017 |
| g_Eisenbergiella | 6.08E-05 | 0.0000 | 0.002 | 0.018 |
| g_Comamonas | 2.67E-05 | 0.0000 | 0.003 | 0.025 |
| f_Prevotellaceae;g_uncultured | 0.0007 | 0.0000 | 0.003 | 0.026 |
| g_Flavonifractor | 0.0008 | 0.0002 | 0.003 | 0.028 |
| g_Eggerthella | 0.0002 | 4.25E-05 | 0.004 | 0.032 |
| g_Faecalitalea | 6.34E-05 | 0.0000 | 0.005 | 0.038 |
| g_Anaerotruncus | 4.22E-05 | 7.08E-06 | 0.006 | 0.043 |
| g_Coprobacter | 0.0004 | 5.39E-05 | 0.007 | 0.053 |
| g_Fenollaria | 9.45E-06 | 0.0000 | 0.008 | 0.056 |
| g_Hungatella | 0.0004 | 0.0000 | 0.0089 | 0.058 |
| g_Erysipelatoclostridium | 0.0004 | 0.0000 | 0.011 | 0.068 |
| g_Family_XIII_AD3011_group | 3.1E-05 | 0.0000 | 0.012 | 0.075 |
| g_Prevotellaceae_Ga6A1_group | 0.0005 | 0.0000 | 0.015 | 0.086 |
| g_Frisingicoccus | 2.41E-05 | 0.0000 | 0.016 | 0.092 |
| g_Raoultella | 6.18E-05 | 0.0000 | 0.019 | 0.106 |
| g_Butyrivibrio | 0.0006 | 0.0000 | 0.035 | 0.188 |
| g_Barnesiella | 0.0022 | 0.0008 | 0.036 | 0.188 |
| g_Rikenellaceae_RC9_gut_group | 2.79E-05 | 0.0000 | 0.039 | 0.192 |
| g_Lachnospiraceae_FCS020_group | 0.0003 | 0.0011 | 0.039 | 0.192 |
| g_Acidaminococcus | 0.0002 | 0.0000 | 0.041 | 0.195 |
| g_Slackia | 1.81E-05 | 0.0000 | 0.044 | 0.203 |

**Supplementary Table 26. Associations of microbiota with protein intake at genus level using t-test**

| **Feature** | **Average Relative Abundance** | | | ***P*-value** | ***Q*-value** |
| --- | --- | --- | --- | --- | --- |
|  | **Meat** | **Bean-products** | |  |  |
| g_Fusobacterium | 0.0088 | 4.77E-05 | | <0.001 | 0.012 |
| g_Sutterella | 0.0074 | 0.0037 | | 0.002 | 0.116 |
| g_Negativibacillus | 0.0003 | 0.0001 | | 0.004 | 0.116 |
| g_Dorea | 0.0068 | 0.0050 | | 0.004 | 0.116 |
| g_Enterorhabdus | 4.76E-05 | 0.0000 | | 0.004 | 0.116 |
| g_Incertae_Sedis | 0.0009 | 0.0005 | | 0.004 | 0.116 |
| g_Mitsuokella | 0.0014 | 0.0002 | | 0.011 | 0.230 |
| g_Butyricimonas | 0.0006 | 0.0003 | | 0.011 | 0.230 |
| g_Anaerotruncus | 5.51E-05 | 1.47E-05 | | 0.021 | 0.386 |
| g_Faecalitalea | 5.42E-05 | 0.0000 | | 0.023 | 0.386 |
| g_Prevotellaceae_NK3B31_group | 0.0018 | 0.0002 | | 0.032 | 0.494 |
| **Feature** | **Average Relative Abundance** | | | ***P*-value** | ***Q*-value** |
|  | **Meat** | | **Dairy** |  |  |
| g_Sutterella | 0.0074 | | 0.0031 | <0.001 | 0.033 |
| g_Fusobacterium | 0.0088 | | 0.0009 | <0.001 | 0.033 |
| g_Mitsuokella | 0.0014 | | 9.1E-05 | 0.003 | 0.193 |
| g_Prevotellaceae_Ga6A1_group | 0.0007 | | 1.46E-05 | 0.010 | 0.334 |
| g_Bifidobacterium | 0.0098 | | 0.0263 | 0.010 | 0.334 |
| g_Christensenellaceae_R-7_group | 0.0035 | | 0.0071 | 0.023 | 0.637 |
| g_Monoglobus | 0.0046 | | 0.0032 | 0.026 | 0.637 |
| g_Lachnoclostridium | 0.0132 | | 0.0097 | 0.036 | 0.667 |
| g_Allisonella | 0.0002 | | 7.94E-05 | 0.039 | 0.667 |
| g_Comamonas | 2.61E-05 | | 4.55E-06 | 0.039 | 0.667 |
| **Feature** | **Average Relative Abundance** | | | ***P*-value** | ***Q*-value** |
|  | **Meat** | | **Eggs** |  |  |
| o_Bacteroidales;f_uncultured;g_uncultured | 8.89E-05 | | 0.0000 | 0.008 | 0.677 |
| g_Lachnospiraceae_UCG-003 | 0.0013 | | 0.0005 | 0.010 | 0.677 |
| g_Sutterella | 0.0074 | | 0.0047 | 0.024 | 0.677 |
| g_Eggerthella | 0.0003 | | 0.0001 | 0.024 | 0.677 |
| g_Romboutsia | 0.0033 | | 0.0021 | 0.025 | 0.677 |
| g_Roseburia | 0.0291 | | 0.0235 | 0.027 | 0.677 |
| g_Alloprevotella | 0.0053 | | 0.0017 | 0.028 | 0.677 |
| g_Dorea | 0.0068 | | 0.0056 | 0.045 | 0.704 |
| g__[Eubacterium]_hallii_group | 0.0104 | | 0.0083 | 0.046 | 0.704 |
| **Feature** | **Average Relative Abundance** | | | ***P*-value** | ***Q*-value** |
|  | **Bean-products** | | **Dairy** |  |  |
| g_Incertae_Sedis | 0.0005 | | 0.0029 | 0.026 | 0.769 |
| g_Intestinimonas | 0.0000 | | 9.77E-06 | 0.027 | 0.769 |
| g_Negativibacillus | 0.0001 | | 0.0005 | 0.027 | 0.769 |
| g_Bifidobacterium | 0.0107 | | 0.0263 | 0.028 | 0.769 |
| g_Bilophila | 0.0007 | | 0.0011 | 0.047 | 0.769 |
| g_Marvinbryantia | 3.7E-05 | | 0.0001 | 0.049 | 0.769 |
| **Feature** | **Average Relative Abundance** | | | ***P*-value** | ***Q*-value** |
|  | **Bean-products** | | **Eggs** |  |  |
| g_Butyricimonas | 0.0003 | | 0.0009 | 0.005 | 0.611 |
| g_Fusobacterium | 4.77E-05 | | 0.0061 | 0.010 | 0.611 |
| g_Incertae_Sedis | 0.0005 | | 0.0011 | 0.013 | 0.611 |
| g_Coprococcus | 0.0097 | | 0.0062 | 0.015 | 0.611 |
| g_Bilophila | 0.0007 | | 0.0012 | 0.024 | 0.664 |
| g_Fusicatenibacter | 0.0055 | | 0.0078 | 0.035 | 0.664 |
| **Feature** | **Average Relative Abundance** | | | ***P*-value** | ***Q*-value** |
|  | **Dairy** | | **Eggs** |  |  |
| g_Colidextribacter | 0.0010 | | 0.0006 | 0.023 | 0.750 |
| g_Fusobacterium | 0.0009 | | 0.0061 | 0.028 | 0.750 |
| g_Bacteroides | 0.2761 | | 0.3330 | 0.030 | 0.750 |

**Supplementary Table 27. Associations of microbiota with dietary preference at genus level using t-test**

| **Feature** | **Average Relative Abundance** | | ***P*-value** | ***Q*-value** |
| --- | --- | --- | --- | --- |
|  | **Balance** | **Vegetables** |  |  |
| g_Enterorhabdus | 4.46E-05 | 0.0000 | 0.003 | 0.260 |
| g_CAG-352 | 0.0064 | 0.0019 | 0.003 | 0.260 |
| g_Holdemanella | 0.0020 | 0.0006 | 0.009 | 0.408 |
| g_Erysipelatoclostridium | 0.0002 | 5.79E-05 | 0.010 | 0.408 |
| g_[Ruminococcus]_gauvreauii_group | 0.0009 | 0.0015 | 0.014 | 0.463 |
| g_Mitsuokella | 0.0016 | 0.0002 | 0.018 | 0.501 |
| g_Eggerthella | 0.0002 | 0.0001 | 0.026 | 0.526 |
| g_Dorea | 0.0063 | 0.0051 | 0.027 | 0.526 |
| g_[Eubacterium]_ventriosum_group | 0.0026 | 0.0018 | 0.031 | 0.526 |
| g_Butyricimonas | 0.0006 | 0.0003 | 0.036 | 0.526 |
| g_RF39 | 3.74E-05 | 7.86E-06 | 0.043 | 0.526 |
| g_Fusicatenibacter | 0.0064 | 0.0088 | 0.043 | 0.526 |
| g_Adlercreutzia | 0.0003 | 0.0002 | 0.046 | 0.526 |
| g_[Ruminococcus]_gnavus_group | 0.0076 | 0.0040 | 0.046 | 0.526 |
| g_Phascolarctobacterium | 0.0073 | 0.0052 | 0.049 | 0.526 |
| **Feature** | **Average Relative Abundance** | | ***P*-value** | ***Q*-value** |
|  | **Balance** | **Meat** |  |  |
| o_Rhodospirillales;f_uncultured;g_uncultured | 0.0014 | 0.0046 | 0.021 | 0.717 |
| g_Fusobacterium | 0.0075 | 0.0028 | 0.027 | 0.717 |
| g_Intestinibacter | 0.0013 | 0.0022 | 0.040 | 0.717 |
| g_Alistipes | 0.0056 | 0.0085 | 0.044 | 0.717 |
| g_Fenollaria | 1.41E-05 | 2.24E-06 | 0.046 | 0.717 |
| **Feature** | **Average Relative Abundance** | | ***P*-value** | ***Q*-value** |
|  | **Vegetables** | **Meat** |  |  |
| g__Holdemanella | 0.0006 | 0.0042 | 0.003 | 0.477 |
| g__Butyricimonas | 0.0003 | 0.0008 | 0.008 | 0.560 |
| g__Dorea | 0.0051 | 0.0069 | 0.010 | 0.560 |
| g_[Eubacterium]_ventriosum_group | 0.0018 | 0.0037 | 0.019 | 0.597 |
| g__Collinsella | 0.0050 | 0.0085 | 0.023 | 0.597 |
| g__CAG-352 | 0.0019 | 0.0075 | 0.023 | 0.597 |
| g__Parabacteroides | 0.0121 | 0.0179 | 0.026 | 0.597 |
| g__Alistipes | 0.0051 | 0.0085 | 0.035 | 0.695 |
| g__Muribaculaceae | 0.0011 | 0.0037 | 0.039 | 0.695 |


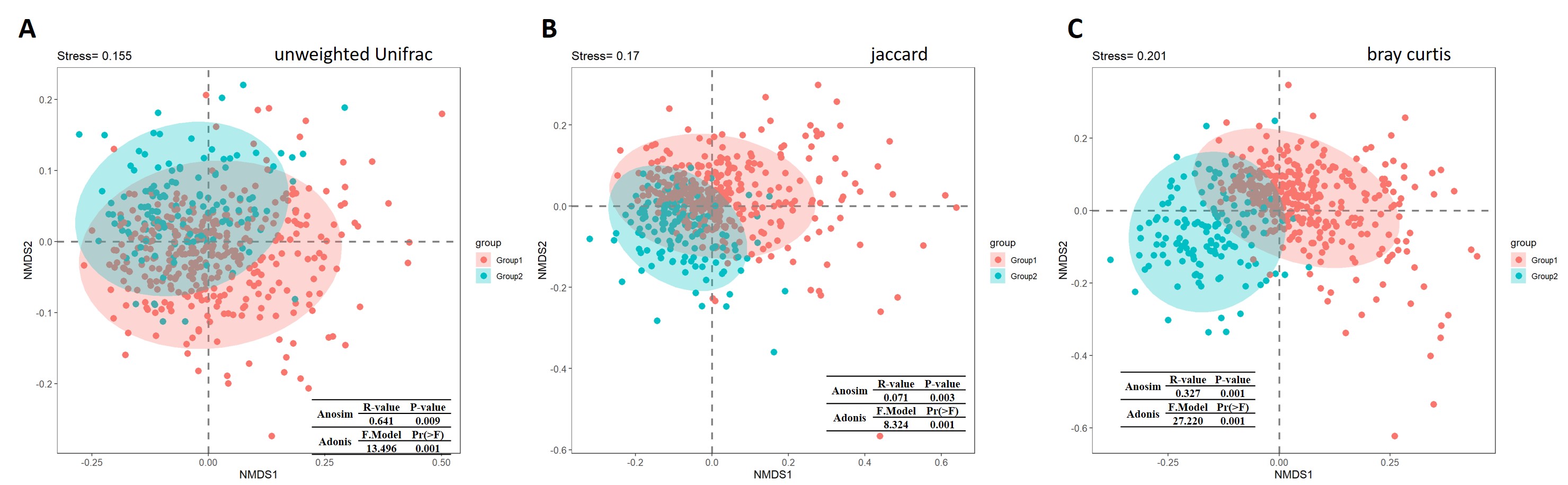


**Supplementary Figure 1. The major enterotypes found in the stool samples from the healthy Chinese population.** Non-metric multidimensional scaling analysis (NMDS) at the ASV level showed two enterotypes (type 1, n = 348; type 2, n = 135). **(A)** NMDS analysis based on unweighted Unifrac distance matric. **(B)** NMDS analysis based on Binary-jaccard distance matric. **(A)** NMDS analysis based on Bray-curtis distance matric.


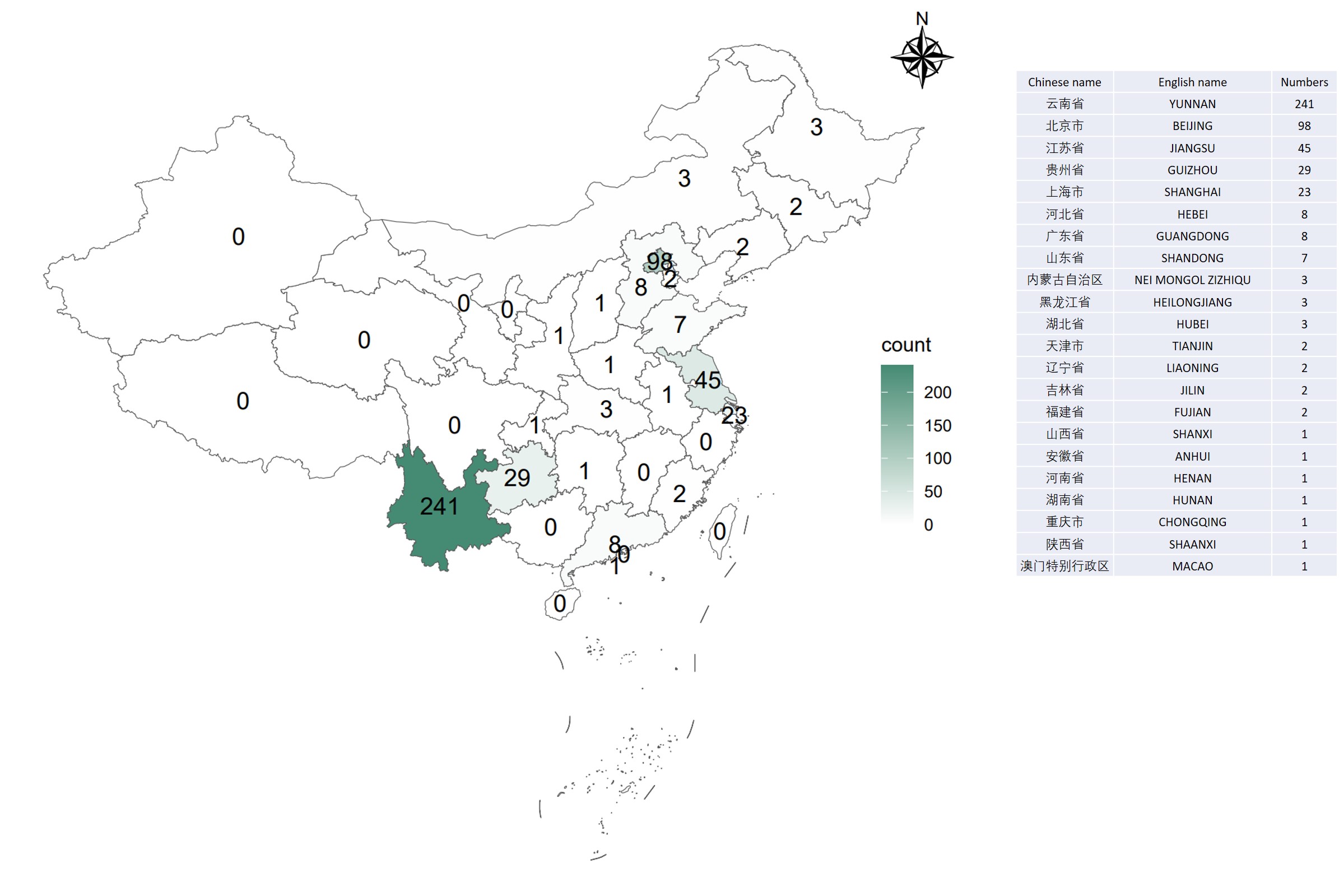


**Supplementary Figure 2. Geographic distribution overview of cohort.** Sampling sites were mapped using sf R package v1.0-13 (https://github.com/r-spatial/sf/) and ggspatial R package v1.1.8 (https://paleolimbot.github.io/ggspatial/) based on Alibaba Cloud DataV.GeoAtlas API (https://geo.datav.aliyun.com/areas_v3/bound/100000_full.json).
